# Integrative Computational Food Toxicology Implicates a CXCL1-Associated Fibroblast Inflammatory Remodeling Signature in Deoxynivalenol-Related Intestinal Barrier Dysfunction

**DOI:** 10.3390/foods15203733

**Published:** 2026-10-20

**Authors:** Haowen Wang, Sungkwon Park, Xuanying Xin, Hangshu Xin, Bin Sun, Xiangzi Li

**Affiliations:** 1 Engineering Research Center of North-East Cold Region Beef Cattle Science and Technology Innovation, Ministry of Education, Yanbian University, Yanji 133002, China; 2 Department of Animal Science, College of Agriculture, Yanbian University, Yanji 133002, China; 3 Department of Food Science and Biotechnology, College of Life Science, Sejong University, Seoul 05006, Republic of Korea; 4 College of Animal Science and Technology, Northeast Agricultural University, Harbin 150030, China

**Keywords:** deoxynivalenol, mycotoxin, food toxicology, intestinal barrier dysfunction, single-cell transcriptomics, CXCL1

## Abstract

Deoxynivalenol (DON) is a prevalent trichothecene mycotoxin with well-recognized intestinal toxicity, yet the molecular and cellular remodeling patterns associated with DON-related intestinal barrier dysfunction remain incompletely understood. Here, an integrative computational framework combining network toxicology, bulk transcriptomics, WGCNA, machine learning, immune infiltration analysis, molecular docking, single-cell transcriptomics, CellChat, and in silico gene perturbation was applied. A total of 1119 distinct genes were obtained from the traceable retained intestinal disease- and barrier-related lists from CTD, GeneCards, OMIM, and PharmGKB. Intersecting this set with differentially expressed genes and genes in the disease-associated WGCNA module yielded 23 database- and transcriptome-supported intestinal disease/barrier candidate genes, which were subsequently evaluated for their potential relevance to DON-related intestinal barrier dysfunction. Machine learning further prioritized six hub genes: SLC6A14, TIMP1, CXCL1, S100A11, LCN2, and NOS2. Single-cell mapping highlighted fibroblasts as an important disease-associated stromal compartment, while CellChat analysis revealed enhanced CXCL signaling in disease samples. CXCL1 showed prominent expression in disease-associated fibroblasts, and its in silico knockout perturbed genes related to inflammatory remodeling, oxidative stress, extracellular matrix organization, and stromal activation. Fibroblast subclustering further identified disease-enriched CXCL-associated inflammatory and stress-response states. Overall, these findings identify CXCL1-associated fibroblast inflammatory remodeling as a candidate stromal context potentially relevant to DON-related intestinal barrier dysfunction, rather than as a DON-specific mechanism, and provide a hypothesis-generating basis for future experimental validation.

## 1. Introduction

Deoxynivalenol (DON), also known as vomitoxin, is a type B trichothecene mycotoxin produced predominantly by *Fusarium graminearum* and *Fusarium culmorum*, two major DON-producing species associated with cereal crops. Owing to the frequent contamination of wheat, maize, barley, and other cereal grains, DON has become a major concern in food and feed safety [1,2]. Risk assessment provides a structured framework for evaluating potential hazards across livestock production and food chains [3]. Regulatory authorities have established commodity-specific limits or advisory levels for DON. For example, Commission Regulation (EU) 2024/1022 sets maximum levels of 1000 μg/kg for most unprocessed cereal grains, 600 μg/kg for cereal milling products, and 400 μg/kg for bakery wares, while the U.S. Food and Drug Administration (FDA) specifies an advisory level of 1 ppm for finished wheat products intended for human consumption [4,5]. Nevertheless, DON contamination remains difficult to completely avoid because of its widespread occurrence in cereals and relative stability during processing [6,7]. Therefore, understanding the biological effects of DON exposure is important for evaluating its toxicological relevance in both food safety and feed safety contexts.

The gastrointestinal tract is one of the primary biological targets of DON after ingestion. Intestinal microbial metabolites and immune-cell trafficking also participate in communication with other organs, as illustrated by the gut–breast axis [8]. Within the intestine, epithelial barrier function, nutrient absorption, and mucosal immune responses are closely interconnected and can be disrupted by DON exposure [9,10]. DON-induced intestinal toxicity should not be regarded as mechanistically independent from its systemic toxicity, because several upstream events, including ribotoxic stress, oxidative stress, MAPK activation, and inflammatory signaling, are shared across tissues. Rather, the intestine represents a distinct toxicological context in which direct luminal exposure to DON can translate these shared molecular insults into barrier-specific consequences, including disruption of epithelial junctions, increased mucosal permeability, and altered multicellular homeostasis. Accumulating evidence indicates that DON can impair epithelial barrier function by altering tight junction proteins, including claudin-1, claudin-3, claudin-4, ZO-1, and occludin, although the specific changes vary among experimental models [11,12,13]. More broadly, interactions among gut microbiota, programmed cell death, and macrophage polarization can contribute to inflammatory and metabolic disturbances [14]. However, DON-related intestinal injury is unlikely to be mediated by epithelial cells alone. The intestinal mucosa is composed of diverse epithelial, immune, endothelial, and stromal cell populations that interact dynamically during inflammatory remodeling. Among chemokine-mediated inflammatory pathways, CXCL1 is of particular interest because it is a potent neutrophil chemoattractant acting mainly through CXCR2 and has been implicated in intestinal inflammatory responses [15]. In addition to immune cells, stromal cells such as fibroblasts can contribute to CXCL1 production under inflammatory conditions, thereby linking stromal activation with leukocyte recruitment and mucosal remodeling [16]. However, whether CXCL1-associated fibroblast inflammatory remodeling is involved in DON-related intestinal barrier dysfunction remains unclear. Thus, identifying the molecular networks and cell-type-specific remodeling patterns associated with DON-related intestinal barrier dysfunction is essential for improving our understanding of DON intestinal toxicity.

Inflammatory intestinal diseases provide valuable transcriptomic resources for investigating barrier disruption, mucosal inflammation, oxidative stress, and stromal remodeling. Although inflammatory bowel disease should not be considered equivalent to DON-induced intestinal injury, both conditions share several biological features, including epithelial barrier impairment, immune activation, inflammatory cytokine production, oxidative injury, and altered cell–cell communication within the intestinal microenvironment [17,18,19]. Therefore, integrating a traceable database-derived intestinal disease/barrier gene set with intestinal inflammatory disease transcriptomic datasets may help identify shared molecular signatures and cellular contexts that can be evaluated for their potential relevance to DON-related intestinal barrier perturbation, although potential heterogeneity arising from intestinal tissue site and disease subtype should be carefully considered. Direct high-throughput transcriptomic datasets derived from DON-exposed human intestinal tissues remain scarce; however, publicly available in vitro transcriptomic datasets from DON-exposed human intestinal epithelial cell lines, such as Caco-2 cells, do exist [20]. These cell-line-based datasets were not included in the present study because our integrative framework was designed to investigate tissue-level inflammatory and barrier-remodeling signatures involving epithelial, immune, endothelial, and stromal compartments.

Network toxicology has emerged as a useful strategy for linking environmental toxicants with disease-associated molecular networks. By integrating toxicant-related targets, disease-associated genes, protein–protein interaction networks, pathway enrichment, and computational prediction, network toxicology can provide systematic insight into the potential biological processes affected by toxicant exposure [21]. In parallel, bulk transcriptomic analysis and weighted gene co-expression network analysis can identify disease-related gene modules, whereas machine-learning methods can prioritize candidate biomarkers with diagnostic or biological relevance. However, bulk transcriptomic analysis cannot fully resolve the cellular sources of candidate genes or the intercellular communication patterns involved in tissue remodeling. Single-cell transcriptomic analysis provides an opportunity to map disease-associated genes to specific intestinal cell populations and to characterize cellular heterogeneity within the intestinal mucosa [22]. Ligand–receptor-based methods, such as CellChat, can infer potential cell–cell communication networks from single-cell gene expression data [23]. Complementary computational approaches can reconstruct gene regulatory networks from single-cell transcriptomic data, providing insights into heterogeneous regulatory patterns [24]. Virtual gene knockout methods can then simulate the removal of a target gene from an inferred regulatory network and evaluate predicted changes in gene regulation, providing hypotheses about gene function that require further validation [25].

In this study, we established an integrative computational framework to investigate potential molecular and cellular signatures associated with DON-related intestinal barrier dysfunction. A traceable database-derived intestinal disease/barrier gene set was integrated with differentially expressed genes and weighted gene co-expression network modules derived from intestinal disease transcriptomic datasets. Machine-learning algorithms were applied to identify hub genes, followed by immune infiltration analysis and molecular docking to evaluate their immunological and structural relevance. Single-cell transcriptomic mapping was then performed to localize hub genes across intestinal mucosal cell populations. Furthermore, CellChat-based communication analysis, in silico knockout analysis, and fibroblast subclustering were used to explore disease-associated intercellular communication and stromal remodeling patterns. This study aimed to identify candidate molecular signatures and cell-type-specific remodeling features that may provide hypothesis-generating evidence for future studies of DON-related intestinal barrier dysfunction.

## 2. Materials and Methods

### 2.1. Overall Study Design

**Figure 1:**
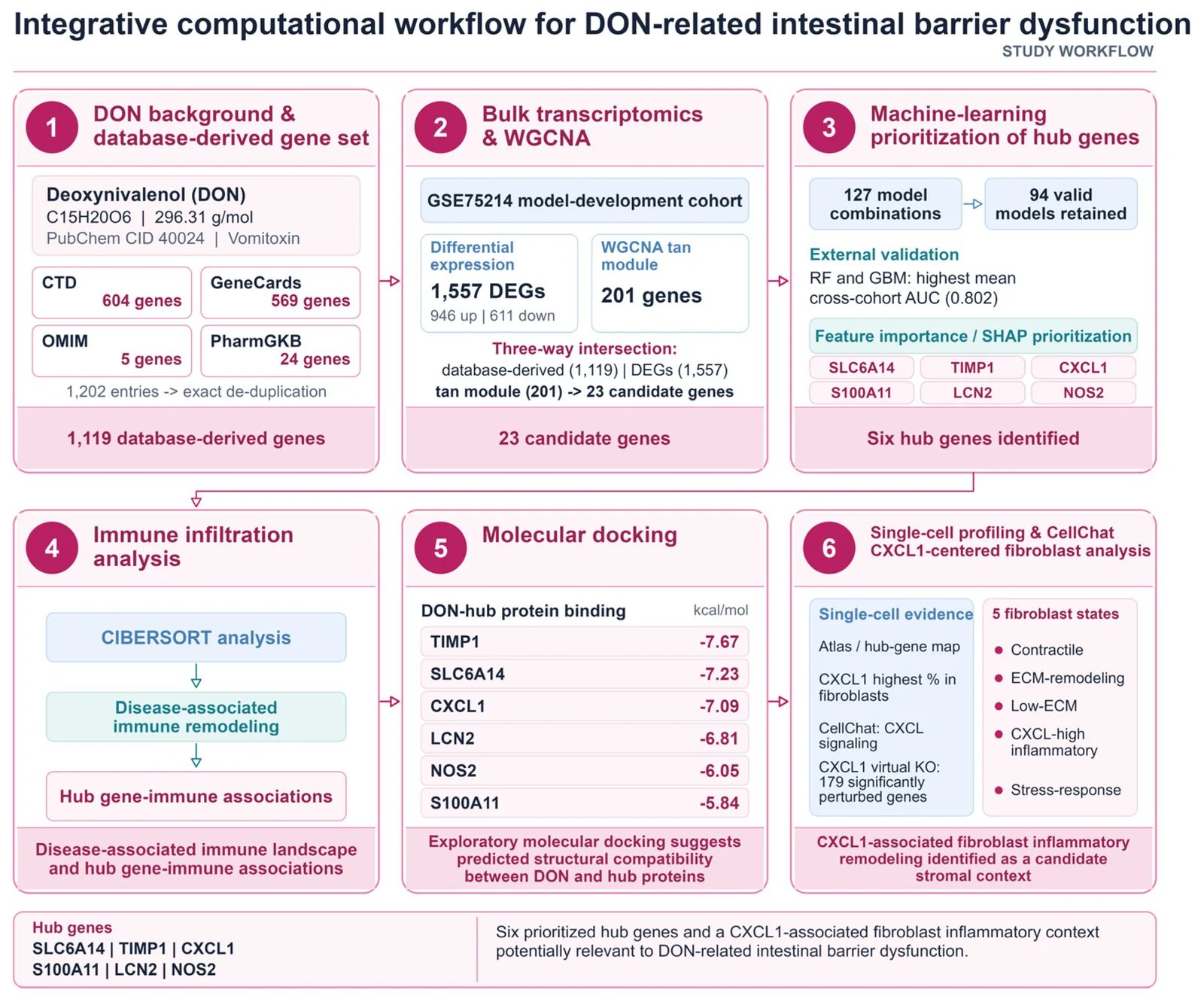
Overview of the integrative computational toxicology framework for DON-related intestinal barrier dysfunction.

This study used an integrative computational framework to identify molecular and cellular features associated with deoxynivalenol (DON)-related intestinal barrier dysfunction (Figure 1). Database-derived genes from CTD, GeneCards, OMIM, and PharmGKB were integrated with differentially expressed genes and disease-associated WGCNA module genes from public intestinal transcriptomic datasets to define candidate genes. Functional enrichment and machine-learning analyses were then used to characterize these candidates and prioritize hub genes, followed by external validation, immune infiltration analysis, and exploratory molecular docking. To resolve the cellular context of the prioritized genes, an independent intestinal single-cell RNA-sequencing dataset was analyzed for cell-type localization, intercellular communication, and fibroblast heterogeneity. Particular attention was given to CXCL signaling and CXCL1, which was further examined using in silico perturbation in disease-associated fibroblasts. Overall, this workflow was designed to generate testable hypotheses regarding DON-related intestinal barrier remodeling rather than to establish direct causal mechanisms.

### 2.2. Transcriptomic Dataset Acquisition and Cohort Definition

Publicly available intestinal transcriptomic datasets were obtained from the Gene Expression Omnibus (GEO) database. Datasets were selected according to the following criteria: (1) intestinal mucosal samples from patients with inflammatory intestinal diseases and non-disease controls were included; (2) gene expression profiles and corresponding sample annotation information were available; (3) UC and/or CD samples were included; and (4) the dataset was suitable for differential expression analysis, co-expression network construction, machine-learning model development, external validation, or single-cell analysis. Datasets derived from non-intestinal tissues, cell-line-only experiments, animal models, or samples lacking clear disease/control annotation were excluded.

GSE75214 was used as the primary model-development cohort. According to the GEO study design, the cohort comprised 194 intestinal mucosal biopsies representing 97 patients with ulcerative colitis (UC), 75 patients with Crohn’s disease (CD), and 11 non-IBD controls. The colon samples included 97 UC, 8 CD, and 11 control biopsies, whereas the terminal ileum samples included 67 CD and 11 control biopsies. The 11 control donors contributed paired colon and ileal biopsies. For the primary comparison, the 172 UC/CD biopsies were combined as the Disease group and the 22 control biopsies were defined as the Control group. GSE75214 was used for differential-expression analysis, WGCNA, candidate-gene screening, machine-learning model development, and immune-infiltration analysis.

GSE193677 was used as an external evaluation cohort. The dataset contained 2490 intestinal biopsies from 1162 participants, including 872 biopsies from 421 patients with UC, 1157 biopsies from 498 patients with CD, and 461 biopsies from 243 controls. Multiple biopsies were available from some participants. The sampled sites included the ileum, cecum, ascending/right colon, transverse colon, descending/left colon, sigmoid colon, and rectum, with both inflamed and non-inflamed biopsies represented. For the Disease-versus-Control evaluation, the 872 UC and 1157 CD biopsies were combined as 2029 Disease samples, whereas the 461 control biopsies were assigned to the Control group.

GSE235236 was used as a second external evaluation cohort. It contained 56 colonic mucosal biopsies from 56 donors, including 26 patients with UC, 22 patients with CD, and 8 healthy controls. The sampled sites included the sigmoid colon (35 biopsies), rectum (9 biopsies), descending colon (6 biopsies), ascending colon (4 biopsies), and transverse colon (2 biopsies). The UC and CD samples were combined as 48 Disease samples, whereas the 8 healthy-control samples were assigned to the Control group.

GSE214695 was used for cell-type-resolved mapping and downstream single-cell analyses. The dataset contained colonic mucosal samples from 18 donors, including 6 healthy controls, 6 patients with UC, and 6 patients with CD. After quality control, 34,556 cells were retained, including 12,416 cells from the Control group and 22,140 cells from the combined UC/CD Disease group. This dataset was used for cell-type annotation, hub-gene localization, cell–cell communication analysis, in silico perturbation analysis, and fibroblast subclustering.

The bulk-transcriptomic analyses were conducted at the biopsy-sample level. In the primary GSE75214 analyses, tissue site was not included as a covariate in the differential-expression, WGCNA, or machine-learning models. Disease subtype and tissue site were partly confounded because all UC samples were colonic, whereas most CD samples were ileal. In GSE193677, multiple biopsies from the same participant were evaluated as individual samples, and neither donor identity nor tissue site was modeled in the cohort-level performance analysis. Consequently, the reported group differences should be interpreted as composite disease-associated transcriptomic patterns that may also reflect tissue-site and within-donor variation.

Detailed information for all included datasets, including GEO accession number, platform, tissue source, disease status, sample or cell number, and analytical role, is summarized in Table 1. All transcriptomic data used in this study were obtained from publicly available databases; therefore, no additional ethical approval or informed consent was required.

### 2.3. Assembly of the Traceable Database-Derived Intestinal Disease/Barrier Gene Set

The archived project files contained four CTD disease-related gene lists labeled “colitis”, “Crohn’s disease”, “inflammatory bowel disease”, and “ulcerative colitis”. Although the intended filtering rule was to retain the top 300 genes ranked by the CTD InferenceScore for each disease term, each archived list contained 299 entries. Because the original CTD exports and intermediate preprocessing files were not retained, the reason for this one-entry discrepancy cannot be reconstructed. After merging the four archived lists and removing exact duplicates, 604 distinct gene symbols were retained.

The two archived GeneCards tables labeled “intestinal disease” and “barrier-related genes” contained 19,029 and 13,249 records, respectively. Genes in each table were ranked in descending order according to the available GeneCards relevance score, and the first 300 records from each table were retained. Their union contained 569 distinct gene symbols after exact deduplication. The retained OMIM and PharmGKB files contributed 5 and 24 genes, respectively. Across the four database sources, 1202 retained entries were combined. Gene symbols were standardized, and exact duplicate, invalid, or unmatched symbols were removed, yielding a traceable set of 1119 distinct genes. Detailed information on the retained database-specific records and filtering procedure is provided in Supplementary Table S1.

Project records indicate that CTD searches using the terms “deoxynivalenol” and “vomitoxin” were performed during an earlier stage of data assembly. However, the original DON-specific exports and the intermediate files documenting their overlap with the intestinal disease-related lists were not retained. Consequently, neither the original DON-specific entry counts nor the subset of the 1119 genes with directly documented DON associations can be reconstructed. The 1119-gene set is therefore treated as a traceable database-derived intestinal disease/barrier gene set rather than as a verified DON-associated gene set. Its downstream intersection with disease transcriptomic signals is interpreted as hypothesis-generating evidence for potential relevance to DON-related intestinal barrier dysfunction, not as evidence of DON-specific regulation.

### 2.4. Differential Expression Analysis

Differential expression analysis between the Disease and Control groups was performed using the limma R package in the model-development cohort. In the primary analysis, UC and CD samples were combined as the Disease group, whereas non-IBD samples were defined as the Control group. Prior to differential expression analysis, gene expression data were preprocessed, normalized, and annotated with official gene symbols. For genes corresponding to multiple probes or duplicated gene symbols, expression values were collapsed to obtain a single representative value for each gene. A linear model with binary disease status as the explanatory variable was fitted to compare gene expression between Disease and Control biopsy samples. Tissue site, disease subtype, and donor identity were not included as covariates in the primary model. The empirical Bayes method implemented in limma was used to moderate the standard errors. *p* values were adjusted for multiple testing using the Benjamini–Hochberg method. Statistically significant differentially expressed genes (DEGs) were defined using Benjamini–Hochberg-adjusted *p* < 0.05 and |log2 fold change (log2FC)| > 0.585. Genes with log2FC > 0 were considered upregulated in the Disease group, whereas genes with log2FC < 0 were considered downregulated. Because tissue site and disease subtype were partly confounded in GSE75214, the resulting coefficients represent unadjusted group differences and cannot be interpreted as tissue-site-independent disease effects. The DEGs were visualized using volcano plots and heatmaps.

### 2.5. Weighted Co-Expression Network Construction

Weighted gene co-expression network analysis (WGCNA) was performed in the model-development cohort to identify disease-associated gene modules. After preprocessing and gene-symbol annotation, the top 8000 genes ranked by median absolute deviation (MAD) were retained. Sample clustering was used to assess potential outliers (Supplementary Figure S2A,B). A signed network was constructed using the WGCNA R package, and a soft-thresholding power of β = 13 was selected based on scale-free topology and mean connectivity (Supplementary Figure S2C). Gene modules were identified using TOM-based hierarchical clustering and dynamic tree cutting. Module eigengenes were then correlated with disease status, with UC and CD samples coded as Disease and non-IBD samples as Control. The tan module showed the strongest positive association with the Disease trait and was retained as the disease-associated module for downstream intersection with differentially expressed genes and the database-derived gene set. Module–trait correlations were calculated using binary Disease/Control status. Tissue site, disease subtype, and donor identity were not included in the module–trait model. The selected module should therefore be interpreted as associated with the unadjusted Disease-versus-Control comparison rather than as a tissue-site-independent disease module.

### 2.6. Candidate Gene Intersection and Pathway Enrichment Analysis

To prioritize intestinal disease/barrier-associated candidate genes, three traceable layers of evidence were integrated: transcriptome-level differential expression, co-expression module membership, and database-derived intestinal disease/barrier relevance. Statistically significant DEGs from the Disease-versus-Control comparison were first intersected with genes in the disease-associated tan module identified by WGCNA. This intersection represented disease-related transcriptomic signals supported by both differential-expression and co-expression evidence. The resulting genes were then intersected with the traceable database-derived intestinal disease/barrier gene set to obtain database- and transcriptome-supported intestinal disease/barrier candidate genes. These candidates were subsequently evaluated for their potential relevance to DON-related intestinal barrier dysfunction. This intersection-based procedure does not establish direct DON responsiveness or DON specificity. The relationships among the three gene sets were displayed using a three-way Venn diagram generated using the WeiShengXin online platform (https://www.bioinformatics.com.cn/). Functional annotation of the inferred candidate genes was performed using the clusterProfiler R package (version 4.21.1). Human gene annotation was based on org.Hs.eg.db (version 3.23.1). Before enrichment analysis, gene symbols were mapped to Entrez Gene identifiers using the bitr function. Gene Ontology (GO) enrichment was conducted across all three GO categories, including biological process, cellular component, and molecular function, by setting ont = “ALL” in enrichGO. Kyoto Encyclopedia of Genes and Genomes (KEGG) pathway enrichment was conducted using enrichKEGG with the organism parameter set to human (organism = “hsa”). The Benjamini–Hochberg method was used for multiple-testing adjustment. Enrichment terms were retained using the default clusterProfiler thresholds of pvalueCutoff = 0.05 and qvalueCutoff = 0.2. Significant GO terms and KEGG pathways were visualized with bubble plots to summarize the major biological processes and pathways represented by the inferred candidate genes.

### 2.7. Integrated Machine-Learning Framework for Hub Gene Prioritization

All 23 candidate genes identified in Section 2.6 were used as the initial predictor set, and GSE75214 served as the model-development cohort. A total of 127 classifier configurations derived from 11 algorithms were evaluated, including support vector machine, LASSO, glmBoost, random forest, stepwise generalized linear model, ridge regression, linear discriminant analysis, elastic net, XGBoost, gradient boosting machine, and naive Bayes. Models that failed during fitting, feature selection, prediction, or performance estimation were excluded, leaving 94 valid configurations.

In the original workflow, the classifier configurations were ranked according to the arithmetic mean of their AUCs across GSE75214, GSE193677, and GSE235236. Therefore, GSE193677 and GSE235236 informed model ranking and were not untouched independent validation cohorts. We consequently refer to them as external evaluation cohorts. Model performance was reported separately for each cohort to show cross-cohort heterogeneity.

The random forest (RF) classifier ranked first under the original cross-cohort ranking procedure and was selected as the final model. The 23 candidate genes were ranked according to RF-derived variable importance. The six highest-ranked features, SLC6A14, TIMP1, CXCL1, S100A11, LCN2, and NOS2, were retained as the final gene panel. SHapley Additive exPlanations (SHAP) analysis was subsequently applied for post hoc model interpretation and did not contribute to feature selection.

### 2.8. Model Interpretation

SHapley Additive exPlanations (SHAP) analysis was applied to the selected six-feature RF classifier as a post hoc interpretability approach. SHAP values were calculated to quantify the contribution of each retained gene to Disease-versus-Control classification at both global and individual-sample levels.

Global feature importance was evaluated by ranking the six genes according to their mean absolute SHAP values. SHAP summary and dependence plots were used to visualize the magnitude and direction of gene-specific contributions. A representative force plot was generated to illustrate how individual genes influenced a sample-level prediction. SHAP analysis was not used to select the six genes.

### 2.9. Immune Cell Profiling

To characterize immune microenvironmental alterations associated with DON-related intestinal barrier dysfunction, immune cell composition was estimated in the model-development cohort using the Cell-type Identification By Estimating Relative Subsets Of RNA Transcripts (CIBERSORT) algorithm. The normalized bulk transcriptomic expression matrix was used as the input file, and the LM22 leukocyte signature matrix was applied to infer the relative abundance of 22 human immune cell populations in each sample. CIBERSORT was run with default parameters, and only samples with a deconvolution *p* < 0.05 were retained for subsequent immune infiltration analyses.

The relative fractions of immune cell populations were compared between the Disease and Control groups to identify disease-associated immune infiltration patterns. The overall immune cell composition was visualized using stacked bar plots and violin plots. To explore relationships among different immune cell populations, Pearson correlation analysis was performed based on the estimated immune cell fractions, and the resulting immune cell–immune cell correlation matrix was displayed as a heatmap.

To further investigate the potential immunological relevance of the machine-learning-prioritized hub genes, correlations between hub gene expression and immune cell fractions were evaluated. Spearman rank correlation analysis was performed between the expression levels of SLC6A14, TIMP1, CXCL1, S100A11, LCN2, and NOS2 and the relative abundance of each immune cell type. The hub gene–immune cell correlation patterns were visualized using correlation heatmaps and scatter plots.

### 2.10. Molecular Docking Analysis

Molecular docking was performed to explore the potential structural compatibility between DON and proteins encoded by the six machine-learning-prioritized hub genes. The three-dimensional structure of DON was obtained from PubChem, while protein structures were retrieved from the Protein Data Bank (PDB) when available or from the AlphaFold Protein Structure Database when experimentally resolved structures were unavailable. Protein and ligand structures were prepared using standard procedures, and docking was performed using known or predicted binding pockets; global docking was used when no suitable pocket information was available.

Docking poses were ranked according to predicted binding energy, with lower values indicating more favorable predicted interactions. Representative binding conformations and key non-covalent interactions were visualized for comparative interpretation. Docking was used only as an exploratory, hypothesis-generating analysis. The predicted interactions do not demonstrate direct target engagement, biological regulation, or causal involvement in DON-related intestinal barrier dysfunction and should therefore be interpreted cautiously.

### 2.11. Single-Cell Quality Control, Doublet Removal, Decontamination, and Cell Type Annotation

The single-cell RNA sequencing dataset GSE214695 was used to investigate the cellular distribution of hub genes and disease-associated intestinal mucosal remodeling patterns. This dataset contained intestinal mucosal samples from healthy controls and patients with inflammatory intestinal diseases. In the main single-cell analysis, healthy control samples were defined as the Control group, whereas UC and CD samples were combined as the Disease group to maintain consistency with the bulk transcriptomic analysis.

Single-cell data processing was performed using the Seurat R package. Raw count matrices were first converted into Seurat objects for each sample. Low-quality cells were removed according to gene number, UMI count, and the percentages of mitochondrial, ribosomal, and hemoglobin-related transcripts. Genes expressed in very few cells and cells with extremely low transcript detection were excluded. Potential doublets were identified and removed before data integration. Ambient RNA contamination was assessed during quality control, and cells with abnormal expression patterns suggestive of contamination or poor quality were excluded from downstream analysis.

After quality control, the retained cells from all samples were merged and subjected to standard preprocessing, including normalization, identification of highly variable genes, scaling, and principal component analysis. Batch effects among samples were corrected using Harmony. The batch-corrected embeddings were then used for neighbor graph construction, unsupervised clustering, and Uniform Manifold Approximation and Projection (UMAP) visualization. Major cell populations were manually annotated according to canonical marker genes and cluster-specific expression patterns. The annotated cell types included epithelial cells, goblet cells, T cells, B cells, plasma cells, monocytes, macrophages, neutrophils, fibroblasts, and endothelial cells. The expression of the six machine-learning-prioritized hub genes, including SLC6A14, TIMP1, CXCL1, S100A11, LCN2, and NOS2, was mapped across the annotated intestinal cell populations.

To further characterize fibroblast heterogeneity, cells annotated as fibroblasts were extracted from the integrated single-cell object and reclustered independently. Fibroblast subclusters were annotated based on subcluster-specific marker genes and functional signatures, including contractile, extracellular matrix-remodeling, inflammatory, stress-response, and low-ECM fibroblast states. The distribution of fibroblast subtypes between the Control and Disease groups was evaluated using cell composition analysis and observed-to-expected ratio enrichment analysis. In addition, the expression patterns of selected hub genes and fibroblast functional module scores were examined across fibroblast subtypes. The module scores included CXCL signaling, inflammatory response, extracellular matrix remodeling, oxidative stress, and myofibroblast contractile signatures.

### 2.12. Cell–Cell Communication Analysis

Cell–cell communication analysis was performed to investigate disease-associated intercellular signaling changes in the intestinal mucosal microenvironment. The annotated single-cell Seurat object was divided into Control and Disease groups according to the group metadata. Cells from healthy control samples were assigned to the Control group, whereas cells from UC and CD samples were combined as the Disease group. Separate Seurat objects were generated for each group and were subsequently used to construct group-specific CellChat objects.

The CellChat R package was used to infer ligand–receptor-mediated communication networks. For each group, the human ligand–receptor interaction database CellChatDB.human was applied. The analysis was focused on secreted signaling interactions by subsetting the database to the Secreted Signaling category. The standard CellChat workflow was then performed for the Control and Disease groups independently, including data subsetting, identification of overexpressed genes and ligand–receptor interactions, communication probability calculation, filtering of low-confidence interactions, pathway-level communication probability inference, network aggregation, and centrality analysis. Low-abundance cell groups with insufficient cell numbers were filtered to reduce unstable communication estimates.

After communication inference was completed for each group, the Control and Disease CellChat objects were merged for comparative analysis. Differences in overall interaction number and interaction strength between groups were evaluated. Differential interaction heatmaps, circle plots, and pathway-level information flow analyses were generated to identify signaling pathways showing disease-associated communication remodeling. In addition, signaling role analysis was performed to characterize the sender, receiver, mediator, and influencer patterns of major cell populations.

Given the machine-learning and single-cell results highlighting CXCL1 and fibroblast-related remodeling, downstream communication analysis focused particularly on CXCL signaling. CXCL signaling networks, communication probability matrices, and signaling role patterns were examined to characterize potential fibroblast-associated inflammatory communication changes in the Disease group. These analyses were used to explore disease-associated intercellular communication patterns and were interpreted as computationally inferred signaling relationships rather than experimentally validated ligand–receptor interactions.

### 2.13. scTenifoldKnk-Based In Silico Knockout Analysis

To explore the potential transcriptional perturbations associated with CXCL1 in disease-associated fibroblasts, in silico single-gene knockout analysis was performed using scTenifoldKnk. Based on the single-cell mapping and CellChat analyses, CXCL1 was selected as the key hub gene for perturbation analysis, and fibroblasts from the Disease group were extracted as the target cell population. This setting was used to evaluate whether virtual CXCL1 knockout could induce downstream transcriptional changes related to inflammatory stromal remodeling.

Before network construction, the fibroblast expression matrix was extracted from the integrated single-cell object. Genes expressed in a sufficient number of cells were retained, and highly variable genes were used for gene regulatory network construction. To avoid excluding the target gene during feature filtering, CXCL1 was retained in the input matrix when present in the fibroblast expression data. The scTenifoldKnk workflow was then applied to simulate CXCL1 knockout and infer transcriptomic perturbations between the original and virtual knockout states.

Differential regulation statistics, including fold change, Z-score, and adjusted q-value, were extracted from the scTenifoldKnk output for downstream interpretation. Perturbed genes were ranked according to fold change and absolute Z-score to identify genes most strongly influenced by virtual CXCL1 knockout. Significant perturbations were defined using the thresholds |Z| ≥ 2 and adjusted q-value < 0.01. The top-ranked perturbed genes were visualized using volcano plots and lollipop plots. Genes affected by CXCL1 virtual knockout were further interpreted in relation to inflammatory microenvironment remodeling, oxidative stress, extracellular matrix organization, and fibroblast activation.

### 2.14. Statistical Analysis and Visualization

All statistical analyses were performed using R software version 4.6.1. Differential expression analysis was conducted using the limma package, and multiple-testing correction was performed using the Benjamini–Hochberg method. Correlations between module eigengenes and disease traits were assessed using Pearson correlation analysis. Associations between hub gene expression and immune cell fractions were evaluated using Spearman rank correlation analysis. Unless otherwise specified, adjusted *p* < 0.05 was considered statistically significant.The R packages used included limma (version 3.69.2), WGCNA (version 1.74), clusterProfiler (version 4.21.1), org.Hs.eg.db (version 3.23.1), Seurat (version 5.5.1), harmony (version 2.0.5), CellChat (version 2.2.0.9001), scTenifoldKnk (version 1.1), ggplot2 (version 4.0.3), pheatmap (version 1.0.13), and ComplexHeatmap (version 2.29.0).Data visualization was performed using ggplot2, pheatmap, ComplexHeatmap, clusterProfiler, Seurat, and CellChat. Venn diagrams were generated using the WeiShengXin online platform (https://www.bioinformatics.com.cn/). Volcano plots, heatmaps, ROC curves, enrichment bubble plots, UMAP plots, dot plots, violin plots, and cell–cell communication networks were generated to summarize the major analytical results.

## 3. Results

### 3.1. Characteristics of the Database-Derived Intestinal Disease and Barrier Gene Set

**Figure 2:**
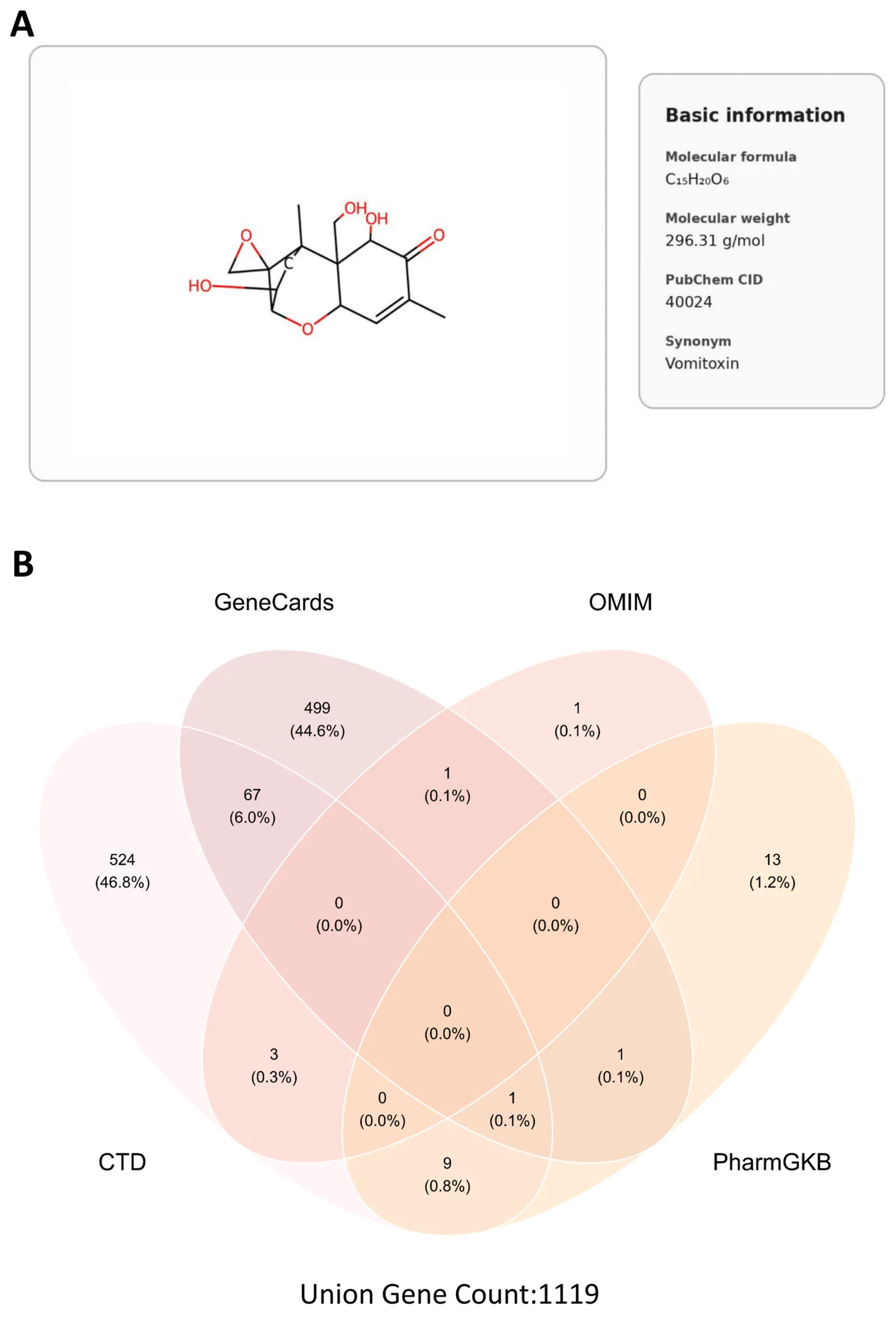
Chemical information of deoxynivalenol (DON) and identification of database-derived intestinal disease gene set. (**A**) The two-dimensional chemical structure and basic chemical information of DON, including molecular formula, molecular weight, PubChem CID, and synonym. (**B**) Venn diagram showing the distribution and overlap of the retained CTD, GeneCards, OMIM, and PharmGKB gene lists. The four lists contained 1202 entries and 1119 distinct gene strings after exact deduplication.

Chemical information for DON, including its two-dimensional structure, molecular formula (C15H20O6), molecular weight (296.31 g/mol), and PubChem CID (40024), is shown in Figure 2A.

The retained database-specific lists contained 604 distinct gene symbols from CTD, 569 from GeneCards, 5 from OMIM, and 24 from PharmGKB. These lists comprised 1202 entries and yielded 1119 distinct gene symbols after exact deduplication (Figure 2B). Of these, 524 occurred only in CTD, 499 only in GeneCards, 1 only in OMIM, 13 only in PharmGKB, and 82 in more than one database. Because the original DON-specific exports and intermediate overlap files were not retained, the 1119-gene set was interpreted as a traceable database-derived intestinal disease/barrier gene set rather than as a verified DON-associated gene set.

### 3.2. Transcriptomic Characterization and Identification of Database- and Transcriptome-Supported Intestinal Disease/Barrier Candidate Genes

Before differential-expression and WGCNA analyses, the overall quality and sample structure of the model-development cohort GSE75214 were evaluated. Expression distributions were broadly comparable across the retained samples, with similar median expression levels and interquartile ranges (Supplementary Figure S1A). Principal component analysis (PCA) showed partial separation between the predefined Disease and Control groups, although substantial overlap remained (Supplementary Figure S1B). When samples were colored by disease subtype, UC, CD, and Control samples showed distinguishable but overlapping distributions (Supplementary Figure S1C). PCA colored by tissue site showed marked separation between colon and ileum samples (Supplementary Figure S1D), indicating that tissue location contributed substantially to the transcriptomic structure. Because tissue site was not included as a covariate in the primary differential-expression, WGCNA, or machine-learning models and was partly confounded with disease subtype, the downstream group differences may reflect both disease-associated and tissue-site-associated variation.

**Figure 3:**
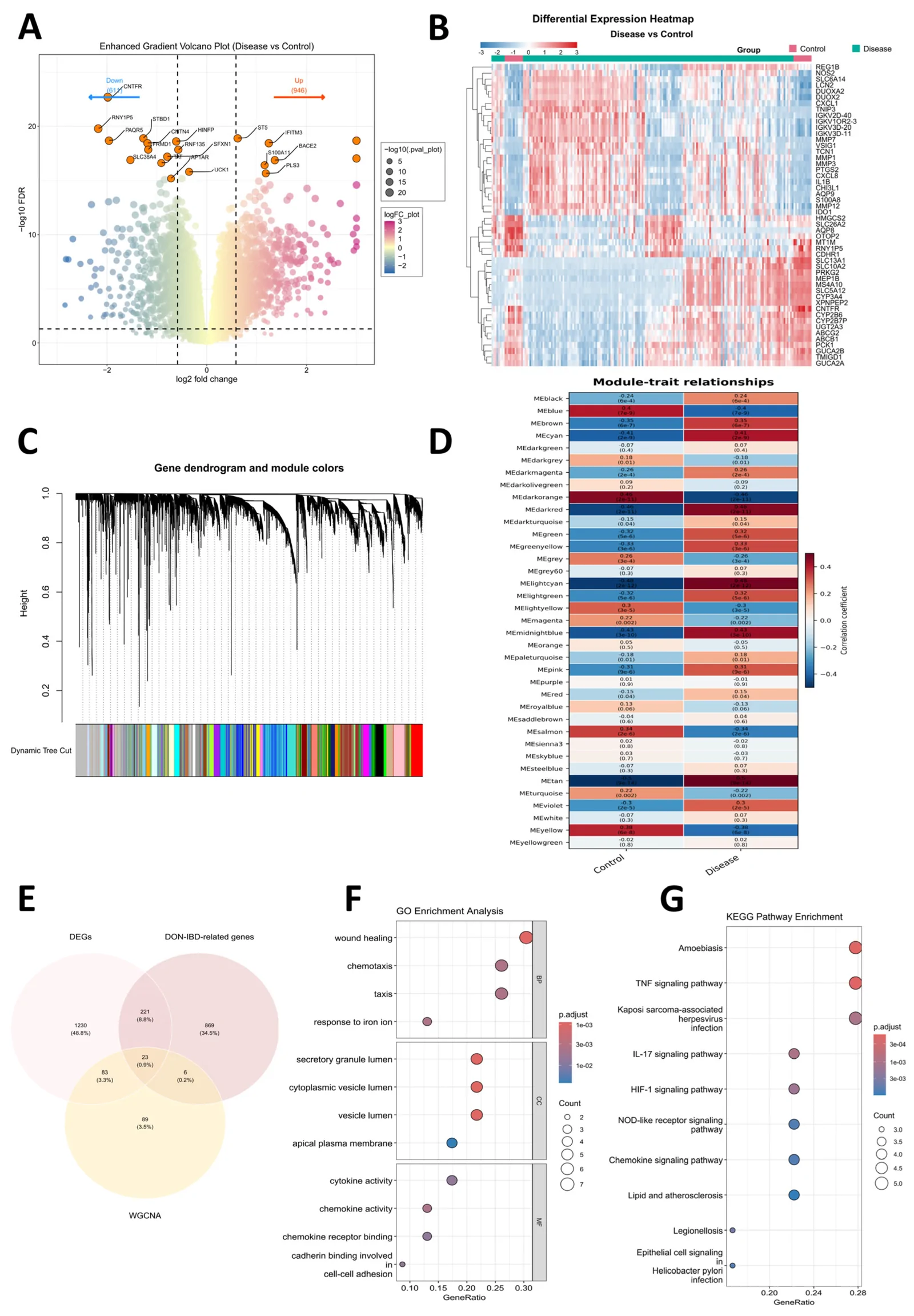
Bulk transcriptomic analysis, WGCNA, candidate gene intersection, and functional enrichment analysis. (**A**) Volcano plot showing differentially expressed genes between Disease and Control samples in the GSE75214 cohort. Red dots indicate upregulated genes, blue dots indicate downregulated genes, and gray dots indicate non-significant genes. (**B**) Heatmap showing the expression patterns of representative differentially expressed genes between the Control and Disease groups. (**C**) Gene dendrogram and module colors generated by WGCNA based on the top 8000 genes ranked by median absolute deviation. (**D**) Module–trait relationship heatmap showing correlations between WGCNA modules and disease status. The tan module showed the strongest positive correlation with the Disease group and was selected as the disease-associated module. (**E**) Venn diagram showing the intersection among differentially expressed genes, the traceable database-derived intestinal disease/barrier gene set, and genes from the disease-associated WGCNA module. A total of 23 overlapping genes were identified as database- and transcriptome-supported intestinal disease/barrier candidate genes and were subsequently evaluated for their potential relevance to DON-related intestinal barrier dysfunction. (**F**) GO enrichment analysis of the 23 candidate genes, including biological process, cellular component, and molecular function categories. (**G**) KEGG pathway enrichment analysis of the 23 candidate genes.

Differential expression analysis was then performed between the Disease and Control groups in GSE75214. Using the thresholds of Benjamini–Hochberg-adjusted *p* < 0.05 and |log2FC| > 0.585, a total of 1557 differentially expressed genes (DEGs) were identified, including 946 upregulated genes and 611 downregulated genes in the Disease group. The global distribution of DEGs is shown in the volcano plot (Figure 3A). A heatmap of the top upregulated and downregulated DEGs showed expression differences between the predefined Disease and Control groups (Figure 3B). However, because tissue site and disease subtype were not included as covariates and were partly confounded, these differences should be interpreted as unadjusted group-associated transcriptomic patterns rather than as tissue-site-independent disease effects.

To identify co-expression modules associated with intestinal disease status, WGCNA was performed using the top 8000 genes ranked by median absolute deviation. Based on scale-free topology assessment, a soft-thresholding power of β = 13 was selected for network construction. Module–trait correlation analysis revealed that the tan module showed the strongest positive correlation with the binary Disease/Control trait (r = 0.50, *p* = 9 × 10^−14^) and was retained as the module most strongly associated with the unadjusted group comparison (Figure 3C,D). Because tissue site was not included in the module–trait analysis, this association may reflect both disease-related and tissue-site-related transcriptional variation.

To further prioritize disease-associated intestinal barrier candidate genes, DEGs, genes in the disease-associated tan module, and the traceable database-derived intestinal disease/barrier gene set were intersected. The three-way Venn analysis identified 23 overlapping genes, which were defined as database- and transcriptome-supported intestinal disease/barrier candidate genes (Figure 3E). These genes were subsequently evaluated as exploratory candidates potentially relevant to DON-related intestinal barrier dysfunction. However, the intersection itself does not establish direct DON responsiveness or DON specificity. GO enrichment analysis indicated that the 23 candidates were mainly involved in immune and inflammatory responses, cytokine-related signaling, leukocyte-associated processes, and extracellular or membrane-associated functions (Figure 3F). KEGG analysis further indicated enrichment of inflammation- and immune-related pathways, supporting their relevance to intestinal inflammatory remodeling and barrier dysfunction (Figure 3G).

Collectively, these analyses identified 23 intestinal disease/barrier candidate genes supported by database-derived evidence, differential expression, and disease-associated co-expression patterns. These candidates provided the basis for subsequent machine-learning prioritization and for hypothesis-generating evaluation in a DON-related intestinal toxicological context.

### 3.3. Machine-Learning Prioritization and SHAP Interpretation of Hub Genes

**Figure 4:**
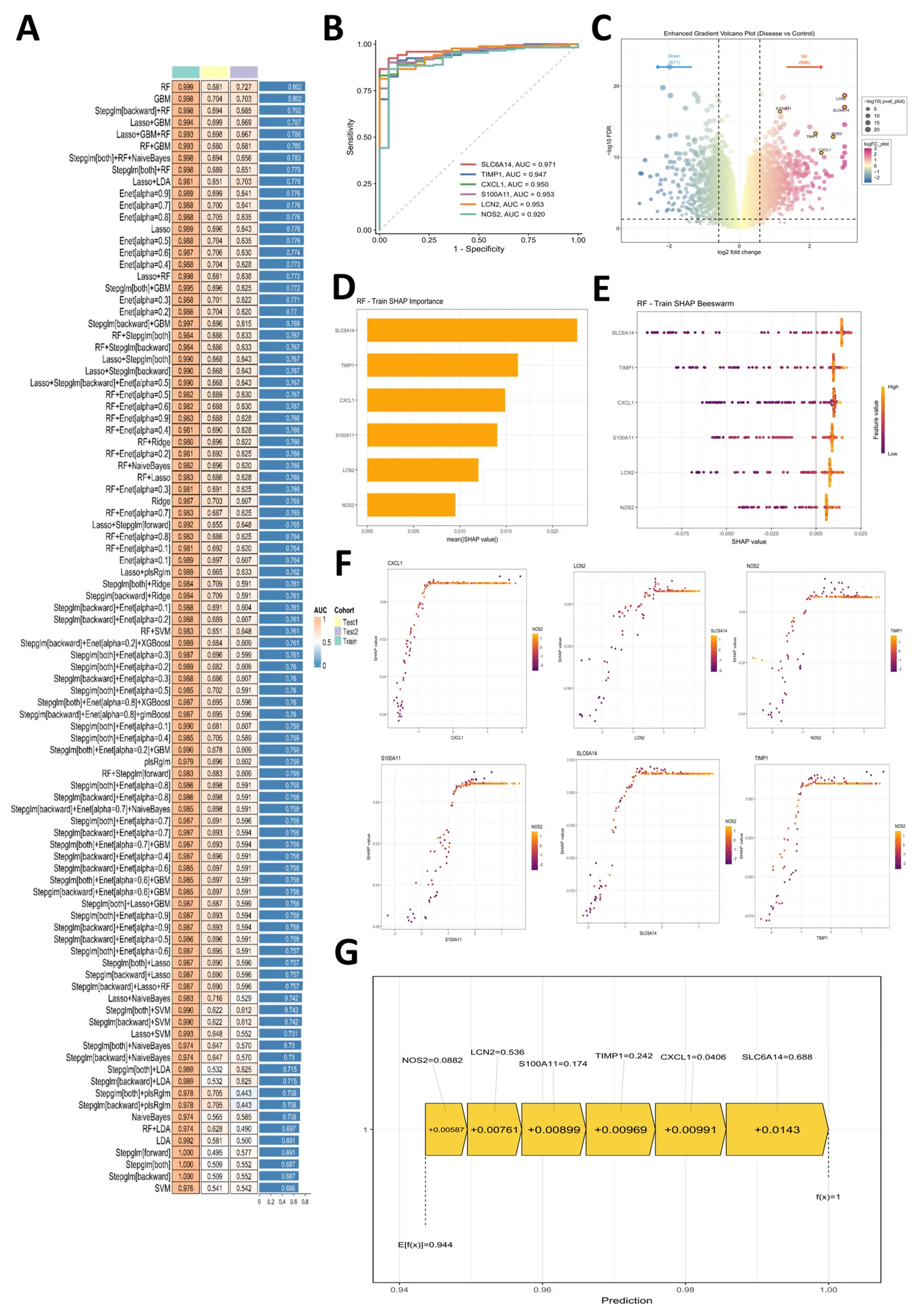
Machine-learning prioritization and interpretation of the six-gene panel. (**A**) Cohort-specific AUC heatmap of the successfully evaluated classifier configurations in GSE75214, GSE193677, and GSE235236. GSE75214 was the model-development cohort, whereas GSE193677 and GSE235236 were external evaluation cohorts. (**B**) Single-gene ROC curves for the six retained genes in GSE75214. (**C**) Volcano plot showing the differential expression of the six retained genes. (**D**) SHAP feature-importance plot for the selected RF classifier. (**E**) SHAP bee swarm plot. (**F**) SHAP dependence plots for the six genes. (**G**) Representative SHAP force plot showing the contribution of each gene to an individual prediction.

An integrated machine-learning framework was used to further prioritize hub genes from the 23 database- and transcriptome-supported intestinal disease/barrier candidate genes. Among the 127 initially considered candidate model combinations, 94 successfully evaluated models with available AUC values were retained for comparative analysis (Supplementary Table S2). The overall AUC distribution of these models is shown in Figure 4A. The top-ranked models included RF, GBM, Stepglm[backward] + RF, Lasso + GBM, and Lasso + GBM + RF (Figure 4B).

Among the 94 successfully evaluated classifier configurations, the random forest (RF) classifier was selected as the final model. The RF classifier achieved an AUC of 0.999 in the model-development cohort GSE75214 and AUCs of 0.681 and 0.727 in the external evaluation cohorts GSE193677 and GSE235236, respectively. Because the two external cohorts contributed to the original model-ranking procedure, they were considered external evaluation cohorts rather than untouched independent validation cohorts. Based on RF-derived variable-importance ranking, the six highest-ranked genes, SLC6A14, TIMP1, CXCL1, S100A11, LCN2, and NOS2, were retained as the final gene panel. The lower AUCs in the external cohorts indicated limited cross-cohort generalizability and possible overfitting.

Single-gene ROC analysis showed high discriminatory performance for all six hub genes in GSE75214, with more variable performance across the two external evaluation cohorts (Supplementary Table S3). All six hub genes were upregulated in the Disease group, with detailed log2FC values provided in Supplementary Table S3. SHAP analysis further characterized the relative contribution of the six hub genes to model prediction at both global and sample-specific levels (Figure 4). Together, these analyses identified six machine-learning-prioritized hub genes for subsequent immune infiltration, molecular docking, and single-cell analyses.

To assess the consistency of the present computational pipeline with established mechanisms of DON intestinal toxicity, several well-characterized barrier- and signaling-related genes were additionally traced across the analytical workflow (Supplementary Table S9). All 11 examined genes, including CLDN1, CLDN3, CLDN4, TJP1, OCLN, EIF2AK2 (PKR), HCK, MAPK1, MAPK3, MAPK8, and MAPK14, were detectable in the GSE75214 expression matrix. Among them, CLDN1, OCLN, and HCK met the predefined differential-expression criteria. CLDN1 was the only examined gene retained in the disease-associated WGCNA tan module and the final 23-gene candidate set, whereas none of these established mechanism-related genes was included among the six machine-learning-prioritized hub genes.

### 3.4. Immune Infiltration Landscape and Hub Gene–Immune Cell Associations

**Figure 5:**
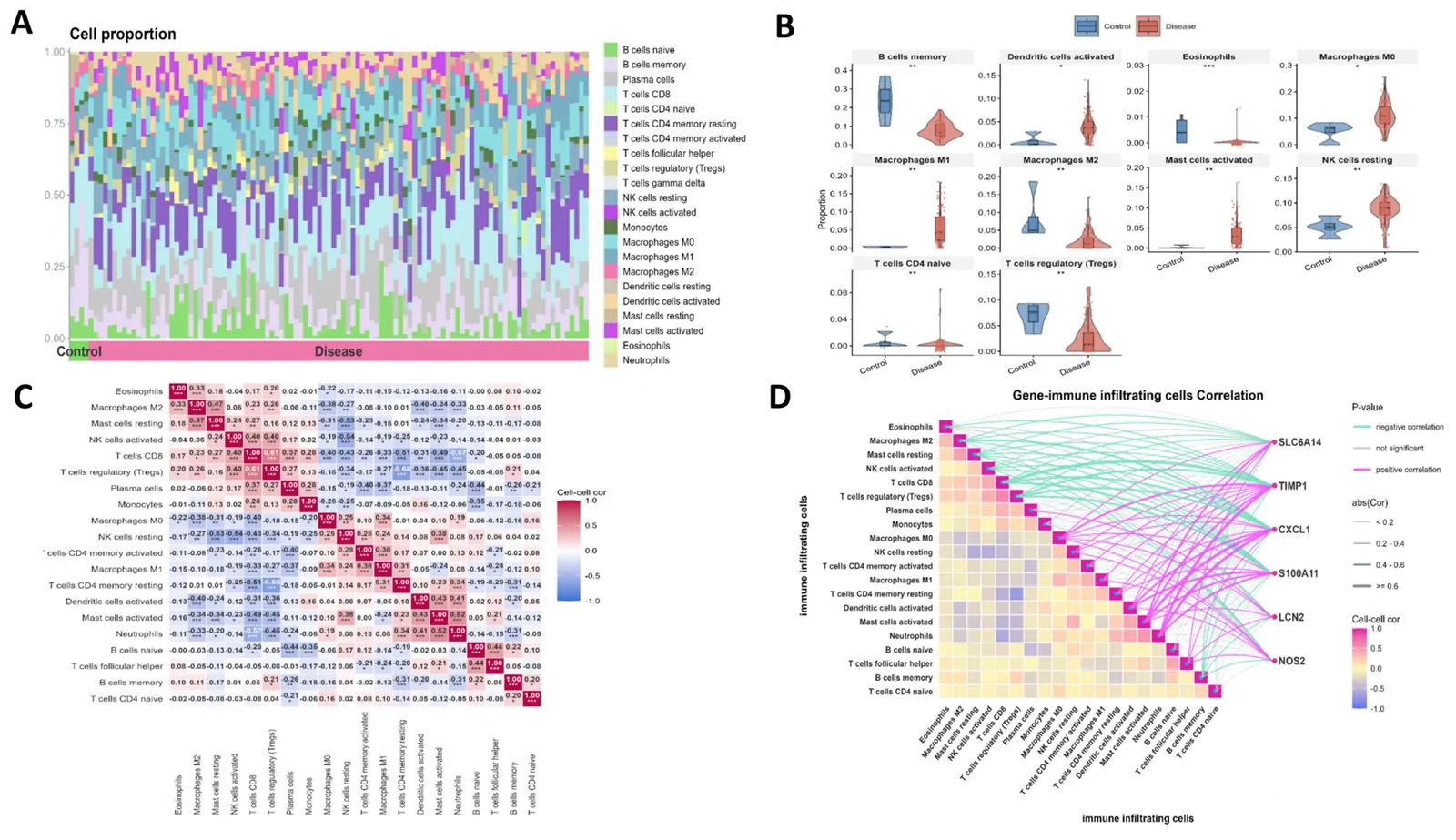
Immune infiltration analysis in the GSE75214 cohort. (**A**) Relative proportions of 22 immune cell populations estimated by CIBERSORT. (**B**) Differentially infiltrated immune cell populations between Control and Disease samples. (**C**) Pairwise immune cell correlation heatmap. (**D**) Correlations between the six hub genes and immune cell fractions. Line color represents correlation direction, and line width represents absolute correlation strength. In panel B, * *p* < 0.05, ** *p* < 0.01, and *** *p* < 0.001 for comparisons between the Control and Disease groups.

CIBERSORT analysis revealed a distinct disease-associated immune infiltration pattern in the GSE75214 cohort (Figure 5A,B; Supplementary Table S4). Compared with the Control group, the Disease group showed increased proportions of CD4 memory resting T cells, resting NK cells, M0/M1 macrophages, activated dendritic cells, activated mast cells, and neutrophils, whereas memory B cells, CD4 naive T cells, Tregs, M2 macrophages, and eosinophils were decreased. These changes indicate a shift toward a more inflammatory immune microenvironment in Disease samples.

Immune cell–cell correlation analysis revealed coordinated co-variation among immune subsets rather than isolated changes (Figure 5C). Tregs were negatively associated with CD4 memory resting T cells, whereas activated mast cells and activated dendritic cells showed positive associations with neutrophils, indicating a structured remodeling of the intestinal immune microenvironment.

Hub gene–immune correlation analysis further linked the six prioritized genes to these disease-associated immune changes (Figure 5D; Supplementary Table S5). Among the increased inflammatory immune subsets, CXCL1 and TIMP1 showed prominent positive correlations with neutrophils, activated mast cells, and activated dendritic cells. In contrast, CXCL1, TIMP1, S100A11, and SLC6A14 were negatively associated with Tregs and M2 macrophages, while memory B cells showed negative correlations with all six hub genes. These findings indicate that the hub-gene signature, particularly CXCL1, was associated with a shift toward a more inflammatory immune microenvironment.

Collectively, these results showed that the Disease group exhibited a distinct immune infiltration pattern characterized by increased innate immune-associated populations and decreased regulatory or repair-associated immune subsets. The six hub genes were broadly correlated with these immune cell changes, particularly involving neutrophils, activated mast cells, activated dendritic cells, Tregs, and M2 macrophages.

### 3.5. Molecular Docking Analysis of DON with Hub-Gene-Encoded Proteins

**Figure 6:**
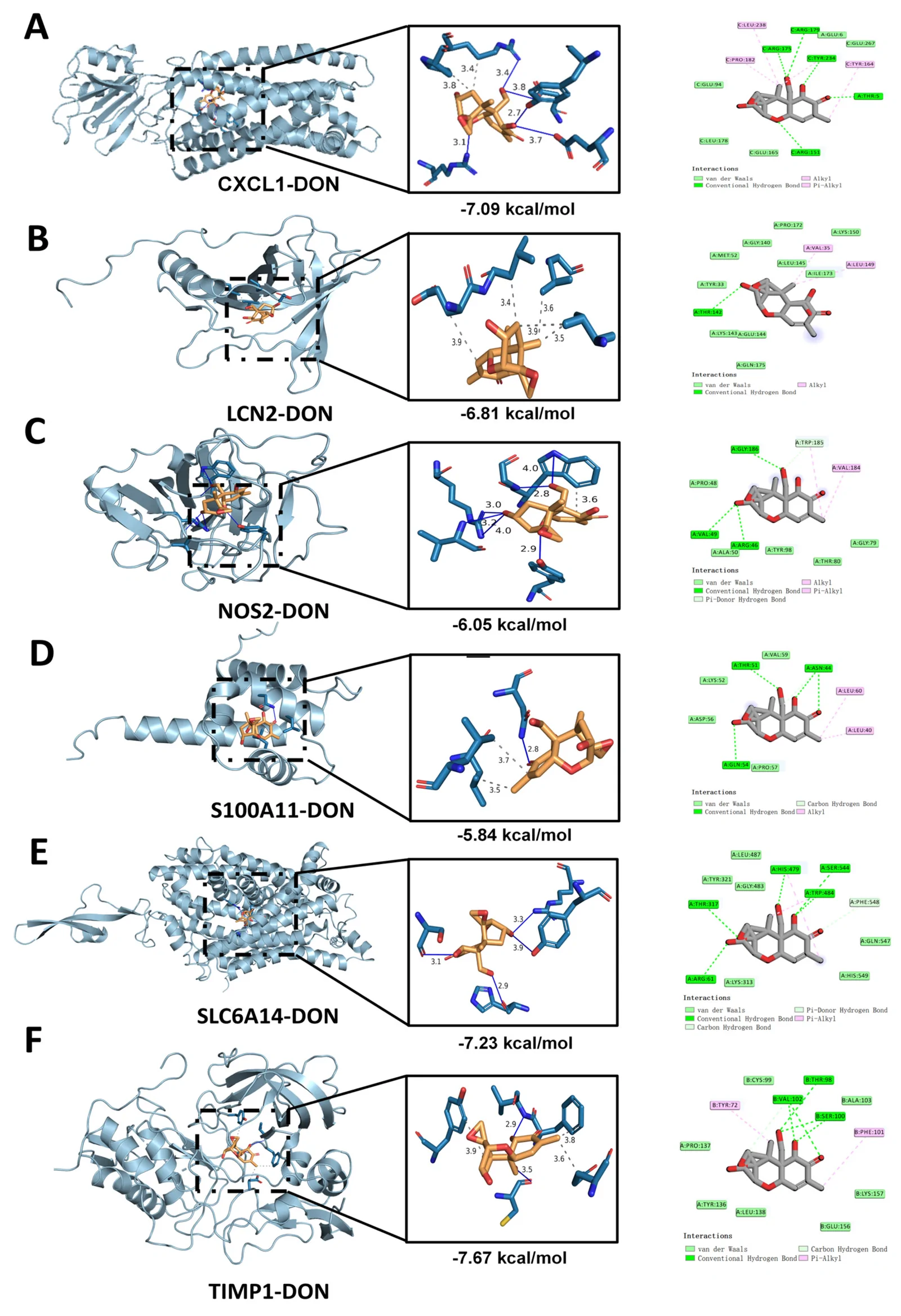
Molecular docking analysis of DON with hub-gene-encoded proteins. (**A**–**F**) Predicted docking conformations, enlarged binding pocket views, and two-dimensional interaction diagrams of DON with CXCL1, LCN2, NOS2, S100A11, SLC6A14, and TIMP1. The predicted binding affinities were −7.09, −6.81, −6.05, −5.84, −7.23, and −7.67 kcal/mol, respectively. The interaction diagrams show the main non-covalent interactions between DON and surrounding amino acid residues.In the two-dimensional interaction diagrams, residue-label colors and dashed-line styles indicate interaction types, as defined in the legend accompanying each panel.

Molecular docking analysis was performed to evaluate the potential structural interactions between DON and the proteins encoded by the six machine-learning-prioritized hub genes, including SLC6A14, TIMP1, CXCL1, S100A11, LCN2, and NOS2. The docking results showed that DON could be accommodated within the predicted interaction regions of all six target proteins and formed multiple non-covalent interactions with surrounding amino acid residues, including hydrogen bonds, van der Waals interactions, hydrophobic contacts, and salt bridge/ionic interactions (Figure 6A–F).

Molecular docking showed negative predicted binding energies for all six DON–protein complexes (Figure 6). Among them, TIMP1, SLC6A14, and CXCL1 exhibited relatively lower binding energies (−7.67, −7.23, and −7.09 kcal/mol, respectively), suggesting more favorable predicted structural compatibility with DON than the other candidate proteins. Representative docking poses indicated that DON could form multiple non-covalent interactions within the predicted binding regions (Figure 6; Supplementary Table S6), including hydrogen bonds, hydrophobic contacts, and van der Waals interactions. These results provide exploratory structural support for the selected hub proteins but should not be interpreted as evidence of direct target engagement or biological causality.

### 3.6. Single-Cell Mapping of Hub Genes in Intestinal Mucosal Cell Populations

**Figure 7:**
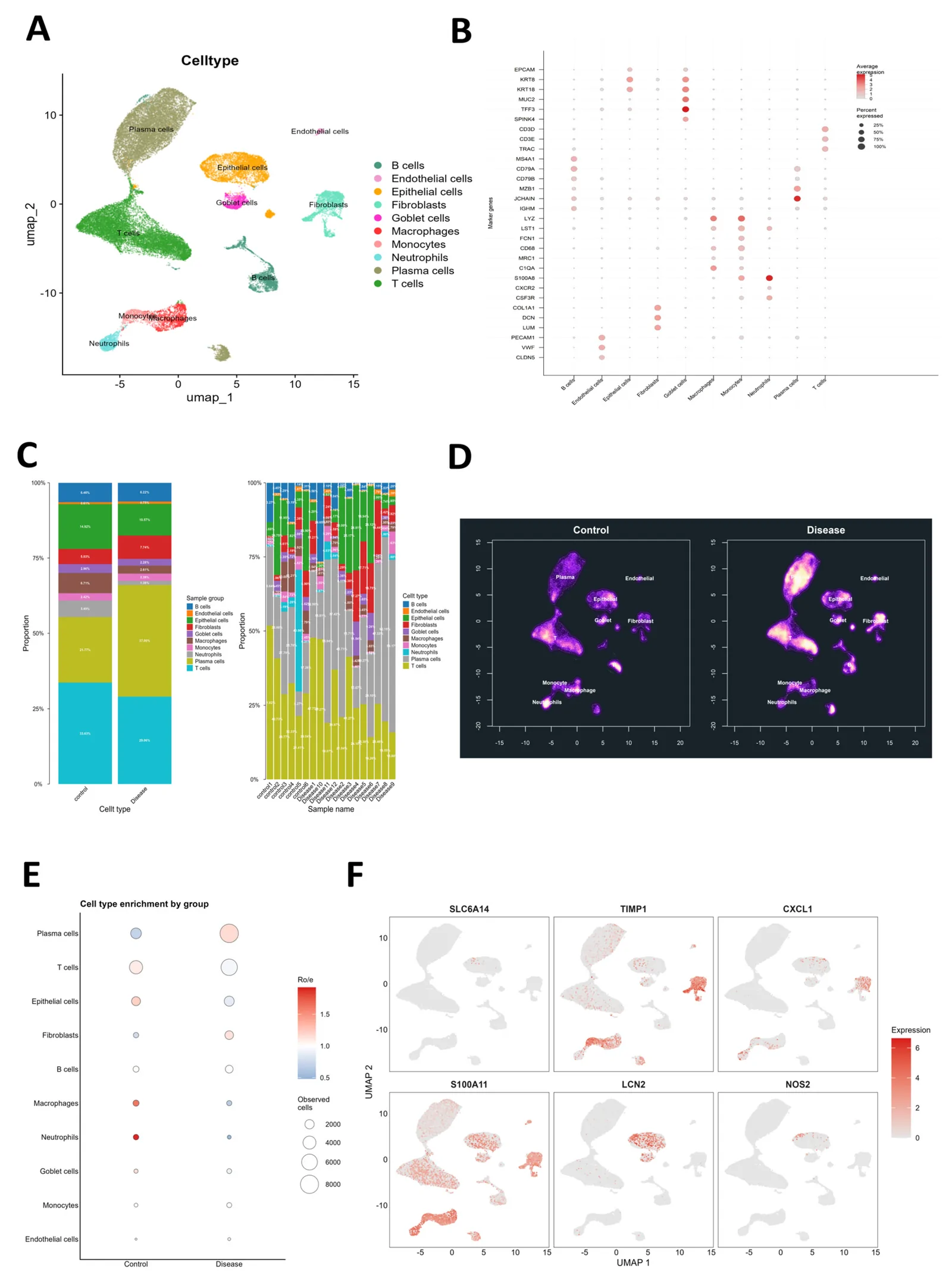
Single-cell mapping of hub genes in intestinal mucosal cell populations. (**A**) UMAP visualization of annotated cell types. (**B**) Dot plot of canonical marker genes used for cell type annotation. (**C**) Cell type composition by group and sample. (**D**) Density distribution of Control and Disease cells on the UMAP space. Brighter colors indicate higher local cell density. (**E**) Cell type enrichment analysis between Control and Disease groups based on observed-to-expected ratios. (**F**) Feature plots showing the expression patterns of SLC6A14, TIMP1, CXCL1, S100A11, LCN2, and NOS2 across intestinal mucosal cells.

Single-cell transcriptomic analysis was performed using the GSE214695 cohort to map the six machine-learning-prioritized hub genes at cellular resolution. After quality control, clustering, and manual annotation, a total of 34,556 intestinal mucosal cells were retained, including 12,416 cells from the Control group and 22,140 cells from the Disease group. These cells were classified into ten major cell populations, including B cells, endothelial cells, epithelial cells, fibroblasts, goblet cells, macrophages, monocytes, neutrophils, plasma cells, and T cells (Figure 7A).

Canonical marker genes were used to validate the cell-type annotations. Epithelial cells were marked by EPCAM, KRT8, and KRT18, while goblet cells expressed MUC2, TFF3, and SPINK4. T cells were characterized by CD3D, CD3E, and TRAC, B cells by MS4A1, CD79A, and CD79B, and plasma cells by MZB1, JCHAIN, and IGHM. Monocytes and macrophages were identified by LYZ, LST1, FCN1, CD68, MRC1, and C1QA, while neutrophils expressed S100A8, CXCR2, and CSF3R. Fibroblasts were characterized by COL1A1, DCN, and LUM, and endothelial cells by PECAM1, VWF, and CLDN5 (Figure 7B).

The cell-type composition analysis showed group-dependent differences between Control and Disease samples (Figure 7C–E; Supplementary Table S7). Plasma cells represented a larger fraction in Disease samples than in Control samples (37.00% vs. 21.77%), and fibroblasts also showed a higher proportion in the Disease group (7.74% vs. 5.03%). In contrast, T cells (29.06% vs. 33.63%), epithelial cells (10.57% vs. 14.92%), macrophages (2.61% vs. 6.71%), and neutrophils (1.38% vs. 5.49%) showed lower proportions in the Disease group than in the Control group. Density mapping on the shared UMAP coordinates further showed differences in the spatial distribution of cells between Control and Disease samples. Observed-to-expected ratio analysis also indicated group-dependent enrichment patterns across the major cell populations, particularly involving plasma cells, fibroblasts, epithelial cells, macrophages, and neutrophils.

The expression patterns of the six hub genes were then mapped onto the single-cell landscape (Figure 7F; Supplementary Table S8). SLC6A14 showed a restricted expression pattern and was mainly detected in epithelial cells. TIMP1 was broadly expressed, with the highest average expression in monocytes, followed by fibroblasts and endothelial cells. CXCL1 showed the highest average expression in neutrophils, whereas the highest expression percentage was observed in fibroblasts. S100A11 was broadly expressed across monocytes, neutrophils, macrophages, endothelial cells, and fibroblasts. LCN2 was mainly enriched in epithelial cells and goblet cells. NOS2 showed relatively sparse expression, with detectable signals mainly in epithelial cells.

### 3.7. Cell–Cell Communication Analysis Highlights CXCL Signaling and CXCL1 Perturbation in Disease Fibroblasts

**Figure 8:**
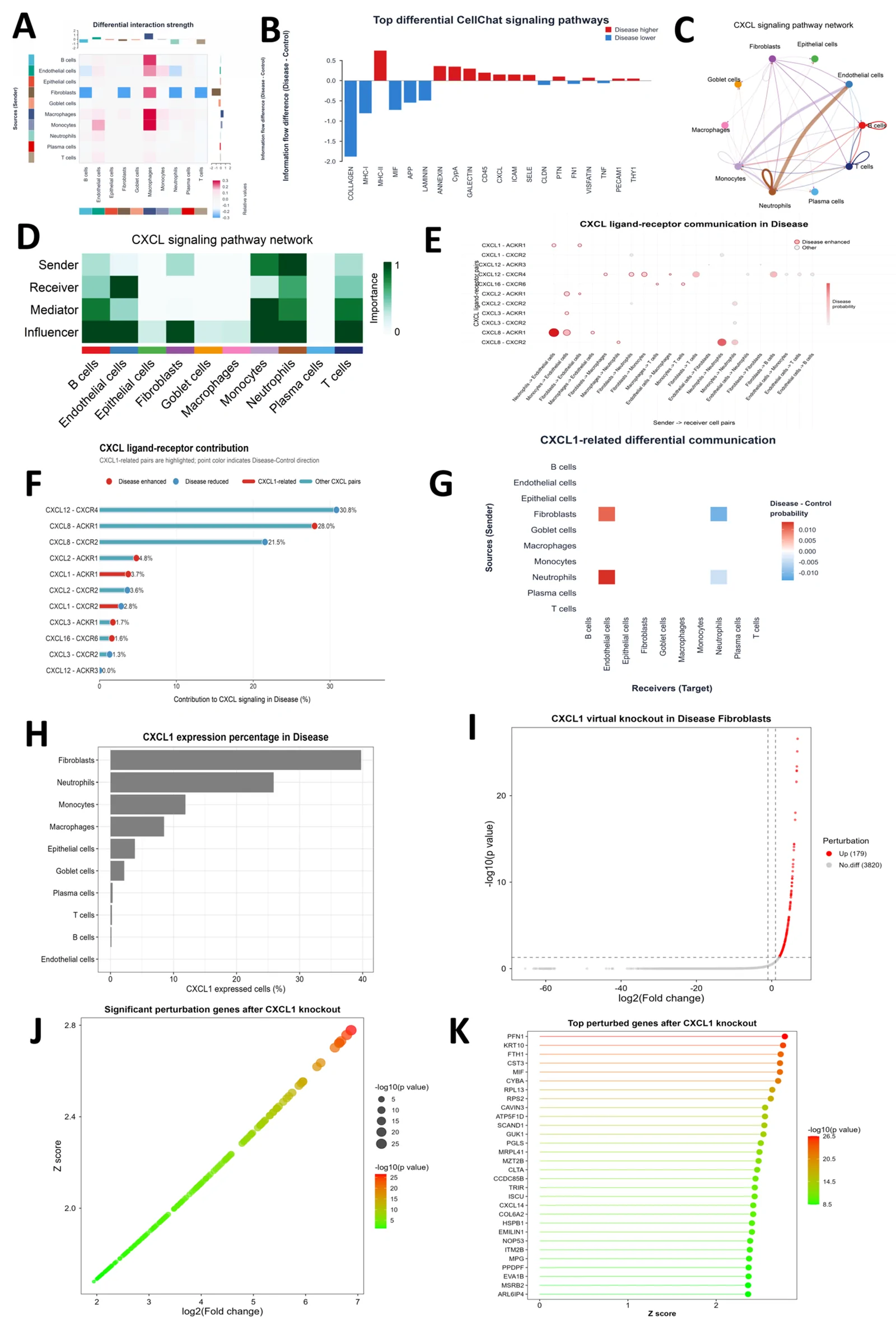
CellChat-based CXCL signaling analysis and CXCL1 virtual knockout in Disease fibroblasts. (**A**) Differential interaction strength among major intestinal mucosal cell populations between Control and Disease groups. (**B**) Differential information flow of CellChat-inferred signaling pathways. (**C**–**G**) CXCL signaling network, signaling roles, ligand–receptor communication patterns, ligand–receptor contributions, and CXCL1-related differential communication. (**H**) Percentage of CXCL1-expressing cells across major cell types in Disease samples. (**I**–**K**) scTenifoldKnk-based CXCL1 virtual knockout analysis in Disease fibroblasts, including perturbed gene distribution and top perturbed genes.

CellChat analysis was performed to characterize intercellular communication remodeling between the Control and Disease groups. Differential interaction analysis revealed marked changes in cell–cell communication strength among major intestinal mucosal cell populations (Figure 8A). Several cell types showed altered outgoing or incoming signaling patterns in the Disease group, indicating extensive remodeling of intercellular communication networks.

Pathway-level CellChat analysis showed increased CXCL signaling information flow in the Disease group (Figure 8B). Within this network, CXCL1-related ligand–receptor interactions, including CXCL1–ACKR1 and CXCL1–CXCR2, were detected and showed disease-associated changes involving multiple intestinal cell populations, including fibroblasts, neutrophils, epithelial cells, and plasma cells (Figure 8C–G).

Cell-type comparison further showed that CXCL1 was most frequently detected in fibroblasts in Disease samples, followed by neutrophils, monocytes, macrophages, epithelial cells, and goblet cells (Figure 8H). This cellular distribution supported the selection of Disease fibroblasts for subsequent CXCL1-focused in silico perturbation analysis.

scTenifoldKnk-based virtual knockout of CXCL1 in Disease fibroblasts identified 179 significantly perturbed genes, whereas 3820 genes showed no significant perturbation (Figure 8I–K). Representative perturbed genes included MIF, CYBA, CXCL14, COL6A2, HSPB1, and EMILIN1, linking the predicted CXCL1-associated transcriptional response to inflammatory remodeling, oxidative stress, extracellular matrix organization, and cellular stress. Together, these findings prioritized CXCL1 as a candidate fibroblast-associated factor within the remodeled CXCL signaling network of Disease samples.

### 3.8. Fibroblast Subclustering Reveals Disease-Associated Fibroblast State Heterogeneity

**Figure 9:**
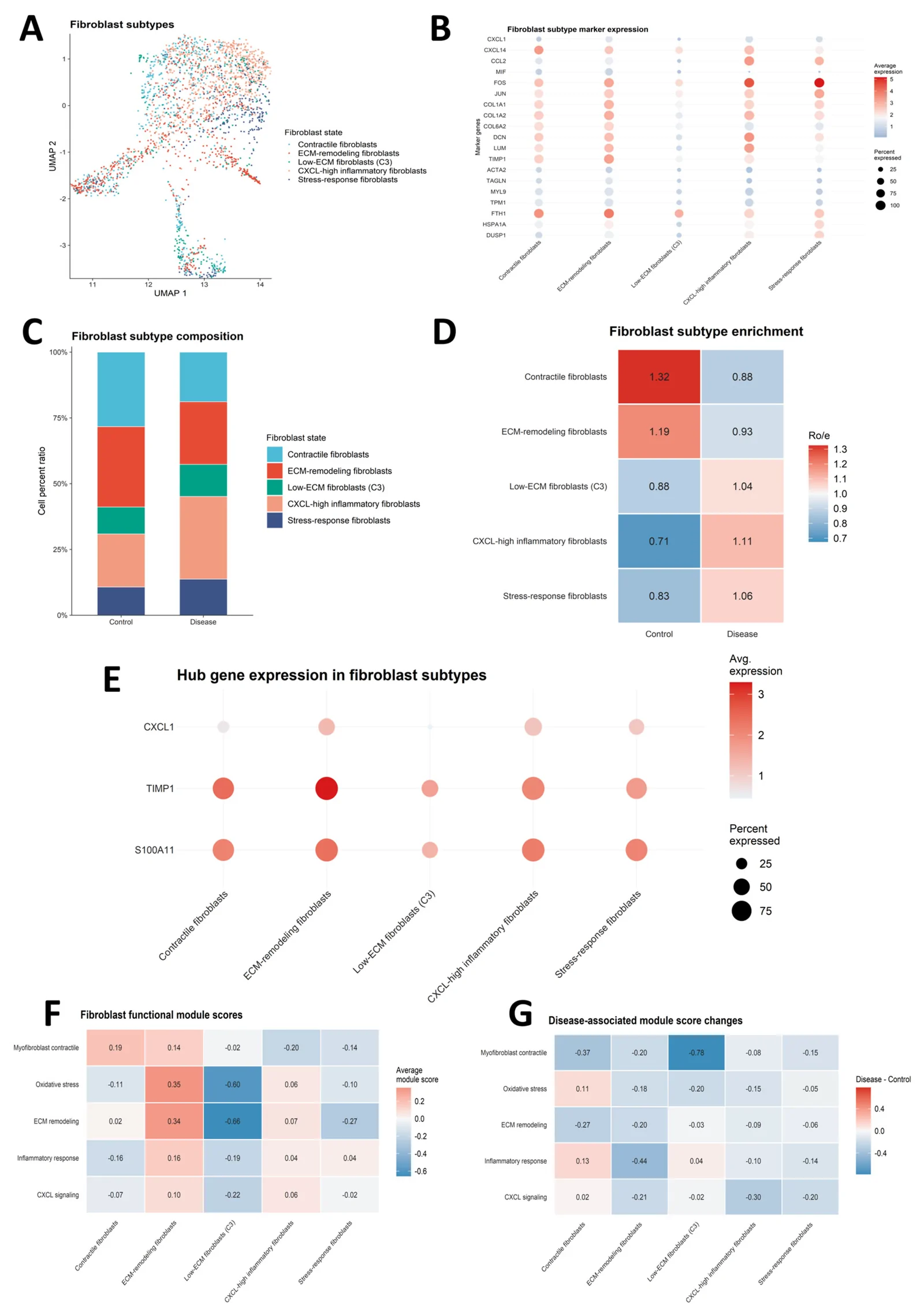
Fibroblast heterogeneity and disease-associated state remodeling. (**A**) UMAP visualization of five fibroblast states. (**B**) Marker gene expression across fibroblast states. (**C**) Fibroblast state composition in Control and Disease groups. (**D**) Observed-to-expected ratio heatmap of fibroblast state enrichment. (**E**) Hub-gene expression across fibroblast states. (**F**) Functional module score patterns across fibroblast states. (**G**) Disease-associated changes in functional module scores.

To further characterize fibroblast heterogeneity, fibroblasts were extracted from the single-cell dataset and reclustered independently. Five fibroblast states were identified, including contractile fibroblasts, ECM-remodeling fibroblasts, low-ECM fibroblasts (C3), CXCL-high inflammatory fibroblasts, and stress-response fibroblasts (Figure 9A). UMAP visualization colored by group showed that both Control- and Disease-derived fibroblasts were distributed across the fibroblast landscape, while Disease fibroblasts were more broadly represented in several fibroblast regions (Supplementary Figure S4A).

Marker expression supported the annotation of five fibroblast states (Figure 9B). Contractile fibroblasts were characterized by ACTA2, TAGLN, MYL9, and TPM1, ECM-remodeling fibroblasts by COL1A1, COL1A2, COL6A2, DCN, LUM, and TIMP1, CXCL-high inflammatory fibroblasts by CXCL1, CXCL14, CCL2, and MIF, and stress-response fibroblasts by FOS, JUN, HSPA1A, DUSP1, and FTH1.

Fibroblast-state composition differed between groups (Figure 9C,D). Disease samples showed greater representation of CXCL-high inflammatory, stress-response, and low-ECM fibroblasts, whereas contractile and ECM-remodeling fibroblasts were relatively more abundant in Control samples. Sample-level variation was also observed (Supplementary Figure S4B).

Among the selected hub genes, CXCL1 was mainly detected in CXCL-high inflammatory and ECM-remodeling fibroblasts, whereas TIMP1 and S100A11 showed broader distributions across fibroblast states (Figure 9E). Functional module scoring further supported these state-specific characteristics: CXCL-high inflammatory fibroblasts showed higher CXCL signaling and inflammatory response scores, ECM-remodeling fibroblasts showed higher ECM-remodeling scores, and contractile fibroblasts showed higher myofibroblast contractile scores (Figure 9F). Together, these results identified a disease-associated shift toward CXCL1-enriched inflammatory fibroblast states.

## 4. Discussion

The toxicological effects of DON have been investigated at several levels, including changes in lymphocyte protein expression [26], experimental nephrotoxicity [27], and population-level dietary exposure through contaminated cereals [28]. Experimental studies have also demonstrated intestinal mucosal injury following exposure to DON and the related mycotoxin nivalenol [29]. In pigs fed diets co-contaminated with DON and zearalenone, changes in oxidative status and responses to antioxidant supplementation have been reported [30]. Therefore, identifying molecular and cellular features associated with DON-related intestinal barrier dysfunction is important for understanding its intestinal toxicological effects. In the present study, we integrated database-derived gene information, bulk transcriptomics, WGCNA, machine learning, and single-cell analyses to characterize molecular and cellular features associated with intestinal barrier dysfunction. Six hub genes, including SLC6A14, TIMP1, CXCL1, S100A11, LCN2, and NOS2, were prioritized. Among them, CXCL1 was further linked to immune infiltration, CXCL signaling, fibroblast expression, and fibroblast transcriptional perturbation, supporting its prioritization as a candidate fibroblast-associated inflammatory context rather than a DON-specific mechanism.

To place our computational findings in the context of established mechanisms of DON intestinal toxicity, we additionally examined several well-characterized barrier- and signaling-related components, including CLDN1, CLDN3, CLDN4, TJP1, OCLN, EIF2AK2 (PKR), HCK, and representative MAPK-family genes. All of these genes were detectable in the GSE75214 expression matrix, and CLDN1, OCLN, and HCK met the predefined Disease-versus-Control differential-expression criteria. Notably, CLDN1 was also retained in the disease-associated WGCNA tan module and the final 23-gene candidate set. However, most of the examined canonical components did not progress through the complete prioritization pipeline. Because GSE75214 represents an intestinal disease comparison rather than direct DON exposure, this analysis should be regarded as a consistency benchmark rather than evidence of DON-induced regulation. The incomplete recovery of canonical DON-related components should therefore not be interpreted as evidence against their established roles. The present workflow prioritizes genes that simultaneously satisfy database-derived inclusion, transcript-level differential expression, and disease-associated co-expression criteria. Consequently, mechanisms mediated predominantly through phosphorylation, translational inhibition, changes in protein abundance or localization, or other post-transcriptional processes may not necessarily emerge as final transcriptomic candidates. Thus, the incomplete recovery of claudin-, PKR/Hck-, and MAPK-related components reflects the scope and limitations of the transcriptome-centered, intersection-based prioritization strategy used here.

The six hub genes identified in this study may represent different aspects of intestinal inflammatory and barrier-related remodeling. SLC6A14 was mainly localized to epithelial cells in the single-cell analysis, suggesting its association with epithelial transport and mucosal metabolic changes. TIMP1 was broadly expressed in monocytes, fibroblasts, and endothelial cells, indicating a potential relationship with extracellular matrix remodeling and stromal activation. In the present analysis, CXCL1 was associated with immune infiltration and CXCL-mediated intercellular communication. This observation is consistent with the broader involvement of CXCL family members in immune-cell recruitment and inflammatory diseases [31]. S100A11 showed broad expression across several immune, stromal, and epithelial cell populations, consistent with its potential involvement in mucosal stress responses. LCN2 was mainly enriched in epithelial cells and goblet cells, suggesting a possible relationship with epithelial injury-associated responses. NOS2 showed relatively sparse expression at the single-cell level but remained part of the machine-learning-prioritized hub-gene panel, indicating that nitric oxide-related inflammatory signaling may also contribute to DON-related intestinal perturbation. However, these genes should be interpreted as computationally prioritized candidate signatures rather than experimentally validated DON-responsive genes.

Immune infiltration analysis provided additional evidence linking the hub-gene signature to the intestinal immune microenvironment. Disease samples showed increased fractions of resting CD4 memory T cells, resting NK cells, macrophages M0, macrophages M1, activated dendritic cells, activated mast cells, and neutrophils, whereas memory B cells, CD4 naive T cells, Tregs, macrophages M2, and eosinophils were decreased. These results indicate that the disease-associated intestinal transcriptomic profile was accompanied by changes in both innate and adaptive immune cell components. The hub genes were also broadly correlated with immune cell fractions. In particular, TIMP1, CXCL1, and S100A11 showed positive correlations with neutrophils, activated mast cells, and activated dendritic cells, while they were negatively correlated with Tregs and macrophages M2. These associations suggest that the hub-gene panel is closely related to immune infiltration remodeling in intestinal disease samples. Nevertheless, immune infiltration analysis based on bulk transcriptomic deconvolution provides correlation-based estimates and does not directly prove immune-cell recruitment or activation.

Molecular docking analysis was used to provide complementary structural information for the relationship between DON and the six hub-gene-encoded proteins. DON showed negative binding energies with all six candidate proteins, with relatively lower binding energies observed for TIMP1, SLC6A14, and CXCL1. Residue-level interaction analysis further showed that DON formed multiple predicted non-covalent interactions, including hydrogen bonds, van der Waals interactions, hydrophobic contacts, and salt bridge or ionic interactions. These results suggest that DON has predicted structural compatibility with these candidate proteins. However, molecular docking should be interpreted with caution because it is based on simplified computational conformations and does not demonstrate direct target engagement, protein activation or inhibition, or biological causality. Therefore, the docking results in this study should be considered supportive structural evidence for hypothesis generation.

Single-cell transcriptomic mapping further clarified the cellular context of the hub genes. The six genes showed distinct localization patterns across epithelial, immune, stromal, and endothelial compartments. SLC6A14 and LCN2 were mainly associated with epithelial-related populations, while TIMP1 and S100A11 were broadly expressed across stromal and immune compartments. CXCL1 showed the highest average expression in neutrophils, whereas its highest expression percentage was observed in fibroblasts. CellChat provides a framework for inferring and comparing cell–cell communication networks across biological conditions [32]. Using this framework, our analysis indicated enhanced inferred CXCL signaling in disease samples. Several CXCL-related ligand–receptor interactions, including CXCL1–ACKR1 and CXCL1–CXCR2, were detected in the communication network. These results linked CXCL1 expression to disease-associated intercellular communication and provided a basis for focusing on fibroblasts in subsequent analyses.

Among the six hub genes, CXCL1 was prioritized as a convergent candidate across multiple analytical layers, including immune infiltration, cell–cell communication, single-cell expression, and fibroblast perturbation analysis. Importantly, CXCL1-associated fibroblast activation is not specific to DON exposure and has been reported in diverse inflammatory conditions. In disease samples, CXCL1 expression was most frequently detected in fibroblasts, followed by neutrophils, monocytes, macrophages, epithelial cells, and goblet cells. Based on this cellular distribution, disease fibroblasts were selected for CXCL1 in silico perturbation analysis. Virtual knockout of CXCL1 in disease fibroblasts resulted in 179 significantly perturbed genes, including MIF, CYBA, CXCL14, COL6A2, EMILIN1, CST3, PFN1, FTH1, and HSPB1. These predicted transcriptional changes can be considered in the broader context of inflammation-associated tissue remodeling. Chronic intestinal inflammation is closely linked to fibrotic remodeling in inflammatory bowel disease [33], while studies of inflammatory joint disease demonstrate that fibroblasts can contribute to inflammatory mediator production and extracellular matrix remodeling [34]. Reactive oxygen species also participate in neutrophil antimicrobial activity, inflammatory tissue damage, and signaling [35]. These processes provide biological context for interpreting the predicted perturbations, although the specific functions of the individual genes in the analyzed intestinal fibroblasts remain to be experimentally established. scTenifoldKnk infers potential gene functions by virtually removing a target gene from a reconstructed gene regulatory network and identifying predicted regulatory changes [36]. Accordingly, the CXCL1 perturbation results obtained here represent computational predictions in disease-associated fibroblasts and require experimental validation. Therefore, these findings should be interpreted as identifying a candidate fibroblast-associated inflammatory context potentially relevant to DON-related intestinal barrier dysfunction, rather than a DON-specific CXCL1 mechanism.

Fibroblast subclustering further supported the presence of disease-associated stromal heterogeneity. Five fibroblast states were identified, including contractile fibroblasts, ECM-remodeling fibroblasts, low-ECM fibroblasts, CXCL-high inflammatory fibroblasts, and stress-response fibroblasts. Disease samples showed higher proportions of CXCL-high inflammatory fibroblasts, stress-response fibroblasts, and low-ECM fibroblasts, whereas contractile and ECM-remodeling fibroblasts were relatively more abundant in Control samples. Marker gene and functional module score analyses further confirmed the functional differences among fibroblast states. CXCL-high inflammatory fibroblasts showed higher CXCL signaling and inflammatory response scores, while stress-response fibroblasts displayed stress-associated features. Together with CellChat and CXCL1 perturbation analyses, these findings support a fibroblast-associated inflammatory remodeling pattern within the analyzed intestinal disease context. Single-cell studies of ulcerative colitis have identified disease-associated inflammatory fibroblast populations [19], and broader studies describe fibroblast heterogeneity and inflammatory functions across multiple disease contexts [37,38]. Accordingly, the fibroblast states identified here should be interpreted as an intestinal inflammatory remodeling context that may be relevant to DON-related barrier dysfunction, rather than as evidence of a DON-specific mechanism.

This study has several limitations. First, the analysis was based on public toxicogenomic resources and public intestinal disease transcriptomic datasets rather than direct DON-exposed intestinal tissue datasets. Therefore, the identified genes should be regarded as database- and transcriptome-supported intestinal disease/barrier candidates that may be relevant to DON-related intestinal dysfunction, rather than as confirmed DON-responsive genes. Second, UC and CD samples were combined into a broad Disease group to capture shared inflammatory and barrier dysfunction-related transcriptional features. However, disease subtype and tissue site were partly confounded in GSE75214 because all UC samples were colonic whereas most CD samples were ileal. Tissue site was not included as a covariate in the primary differential-expression, WGCNA, or machine-learning models, and PCA showed marked colon–ileum separation. Consequently, the identified bulk-transcriptomic signatures may reflect a combination of disease-associated and tissue-site-associated variation. In addition, GSE193677 contained multiple biopsies from some participants, but cohort-level performance was evaluated at the biopsy level without modeling donor identity or tissue site. Because GSE193677 and GSE235236 contributed to the original model-ranking procedure, their AUCs represent external evaluation rather than performance in untouched independent validation cohorts. The reduction in AUC from 0.999 in GSE75214 to 0.681 and 0.727 in the two external cohorts indicates limited model transportability and possible overfitting. These limitations restrict site-independent, donor-independent, and cohort-independent interpretation of the findings. Third, CIBERSORT and CellChat analyses infer immune infiltration and intercellular communication from gene expression data and cannot directly demonstrate cell abundance changes, ligand–receptor activity, or functional signaling [39,40]. Fourth, molecular docking provides computational predictions of protein–ligand interactions [41,42], whereas virtual gene knockout approaches predict regulatory changes following simulated gene removal [25,36]. The predicted DON–protein interactions and CXCL1-associated transcriptional perturbations identified here therefore require experimental validation. More broadly, an important distinction should be made between computationally predicted associations and experimentally demonstrated biological effects. The convergence of these computational findings strengthens the prioritization of CXCL1-associated fibroblast inflammatory remodeling as a testable hypothesis, but does not establish direct DON–protein binding, CXCL1-dependent signaling, fibroblast activation, or causal intestinal barrier injury. Finally, the candidate CXCL1-associated fibroblast inflammatory remodeling context identified here should be validated in DON-exposed intestinal epithelial cells, fibroblast-containing co-culture systems, intestinal organoids, or animal models.

In summary, this study integrated network toxicology, transcriptomics, machine learning, molecular docking, immune infiltration analysis, single-cell mapping, cell–cell communication inference, and in silico perturbation analysis to identify candidate molecular and cellular signatures associated with DON-related intestinal barrier dysfunction. The six hub genes SLC6A14, TIMP1, CXCL1, S100A11, LCN2, and NOS2 were prioritized as exploratory molecular signatures. Further analyses identified CXCL1-associated fibroblast inflammatory remodeling as a candidate stromal context in which DON-associated molecular signals may intersect with established intestinal inflammatory programs. These findings provide a computational toxicology framework for understanding the potential intestinal effects of the food- and feed-borne mycotoxin DON and offer candidate targets and cellular contexts for future experimental validation.

## 5. Conclusions

In conclusion, this study established an integrative computational framework to identify candidate molecular and cellular features potentially relevant to DON-related intestinal barrier dysfunction. Six hub genes, including SLC6A14, TIMP1, CXCL1, S100A11, LCN2, and NOS2, were prioritized through database integration, transcriptomic analysis, WGCNA, and machine learning. Subsequent immune infiltration, molecular docking, and single-cell analyses linked these genes to immune microenvironment remodeling, predicted DON–protein interactions, and distinct intestinal mucosal cell populations. Among them, CXCL1 was further associated with altered CXCL signaling, prominent expression in disease-associated fibroblasts, and predicted transcriptional perturbation following in silico knockout. Fibroblast subclustering further identified disease-associated CXCL-high inflammatory and stress-response states. Overall, these findings identify CXCL1-associated fibroblast inflammatory remodeling as a candidate stromal context potentially relevant to DON-related intestinal barrier dysfunction. This finding should not be interpreted as a DON-specific response, but rather as a hypothesis-generating intersection between Database-derived intestinal barrier evidence considered in a DON-related toxicological context and established inflammatory remodeling programs in the intestinal microenvironment.

## Figures and Tables

**Table 1:** Information on the public transcriptomic datasets used in this study.

GEO Accession	Data Type	Platform	Tissue Source and Sampling Sites	Disease Subtype and Donor Number	Sample/Cell Number Used in This Study	Analytical Role
GSE75214	Bulk transcriptomics; microarray	GPL6244; Affymetrix Human Gene 1.0 ST Array	Endoscopy-derived intestinal mucosal biopsies. Colon: 97 UC, 8 CD, and 11 control biopsies; terminal ileum: 67 CD and 11 control biopsies.	183 donors: 97 UC, 75 CD, and 11 non-IBD controls.	194 biopsies: 172 disease and 22 control biopsies. The 11 controls contributed paired colon and ileal biopsies.	Model-development cohort; differential-expression analysis; WGCNA; candidate-gene screening; machine-learning model development; immune-infiltration analysis.
GSE193677	Bulk transcriptomics; RNA-seq	GPL16791; Illumina HiSeq 2500	Intestinal biopsies from the ileum, cecum, ascending/right colon, transverse colon, descending/left colon, sigmoid colon, and rectum; inflamed and non-inflamed sites were included.	1162 participants: 421 UC, 498 CD, and 243 controls.	2490 biopsies: 872 UC, 1157 CD, and 461 control biopsies. Multiple biopsies were available from some participants.	External evaluation cohort used for cross-cohort model ranking and cohort-specific performance evaluation.
GSE235236	Bulk transcriptomics; RNA-seq	GPL21290; Illumina HiSeq 3000	Colonic mucosal biopsies. Sigmoid colon: 35; rectum: 9; descending colon: 6; ascending colon: 4; transverse colon: 2.	56 donors: 26 UC, 22 CD, and 8 healthy controls.	56 biopsies: 48 disease and 8 control biopsies.	External evaluation cohort used for cross-cohort model ranking and cohort-specific performance evaluation.
GSE214695	Single-cell RNA-seq	GPL18573; Illumina NextSeq 500	Colonic mucosa.	18 donors: 6 UC, 6 CD, and 6 healthy controls.	18 samples; 34,556 cells retained after quality control, including 22,140 disease and 12,416 control cells.	Single-cell analysis cohort; cell-type annotation; hub-gene localization; CellChat analysis; in silico perturbation; fibroblast subclustering.

Abbreviations: CD, Crohn’s disease; UC, ulcerative colitis; WGCNA, weighted gene co-expression network analysis.

## Data Availability

The original contributions presented in this study are included in the article/Supplementary Materials. Further inquiries can be directed to the corresponding authors.

## Supplementary Materials

The following supporting information can be downloaded at: https://www.mdpi.com/article/10.3390/foods15203733/s1, Figure S1: Quality assessment and principal component analysis of the GSE75214 cohort. (A) Expression distribution boxplot of all retained samples, colored by Control and Disease groups. (B) Principal component analysis (PCA) of Control and Disease samples. (C) PCA of samples stratified by disease subtype, including Control, ulcerative colitis (UC), and Crohn’s disease (CD). (D) PCA of samples stratified by tissue site, including colon and ileum; Figure S2: WGCNA sample assessment and soft-thresholding power determination. (A) Hierarchical clustering dendrogram of GSE75214 samples for outlier detection. (B) Sample dendrogram combined with Control/Disease trait annotation. (C) Scale-free topology model fit across different soft-thresholding powers. (D) Mean connectivity across different soft-thresholding powers. These analyses were used to determine the appropriate soft-thresholding power for WGCNA network construction; Figure S3: ROC curves of representative machine-learning models across the model-development and external evaluation cohorts. (A) ROC curves of the top-ranked machine-learning models in the model-development cohort GSE75214. (B) ROC curves of the same models in the external evaluation cohort GSE193677. (C) ROC curves of the same models in the external evaluation cohort GSE235236; Figure S4: Fibroblast distribution and sample-level subtype composition. (A) UMAP distribution of fibroblasts colored by Control and Disease groups. (B) Sample-level composition of five fibroblast states in Control and Disease samples; Table S1: Sources, selection criteria, and traceability of the database-derived intestinal disease/barrier gene set; Table S2: Performance of machine-learning models across the model-development and external evaluation cohorts; Table S3: Summary of the six machine-learning-prioritized hub genes; Table S4: Differences in immune cell fractions between Control and Disease samples; Table S5: Correlations between hub gene expression and immune cell fractions; Table S6: Predicted residue-level interactions between DON and hub-gene-encoded proteins; Table S7: Single-cell composition of major cell types in Control and Disease samples; Table S8: Expression patterns of the six hub genes across major cell types; Table S9: Representation of genes associated with established DON-related mechanisms across the gene-screening stages.
