# Supplementary material for: Integrative Computational Food Toxicology Implicates a CXCL1-Associated Fibroblast Inflammatory Remodeling Signature in Deoxynivalenol-Related Intestinal Barrier Dysfunction
Source: Foods. 2026 Oct 20;15(20):3733. doi: 10.3390/foods15203733 (PMC13704015; doi:10.3390/foods15203733)
Supplement: Supplementary file 1 [file foods-15-03733-s001.zip › Figure S1-4.pdf]

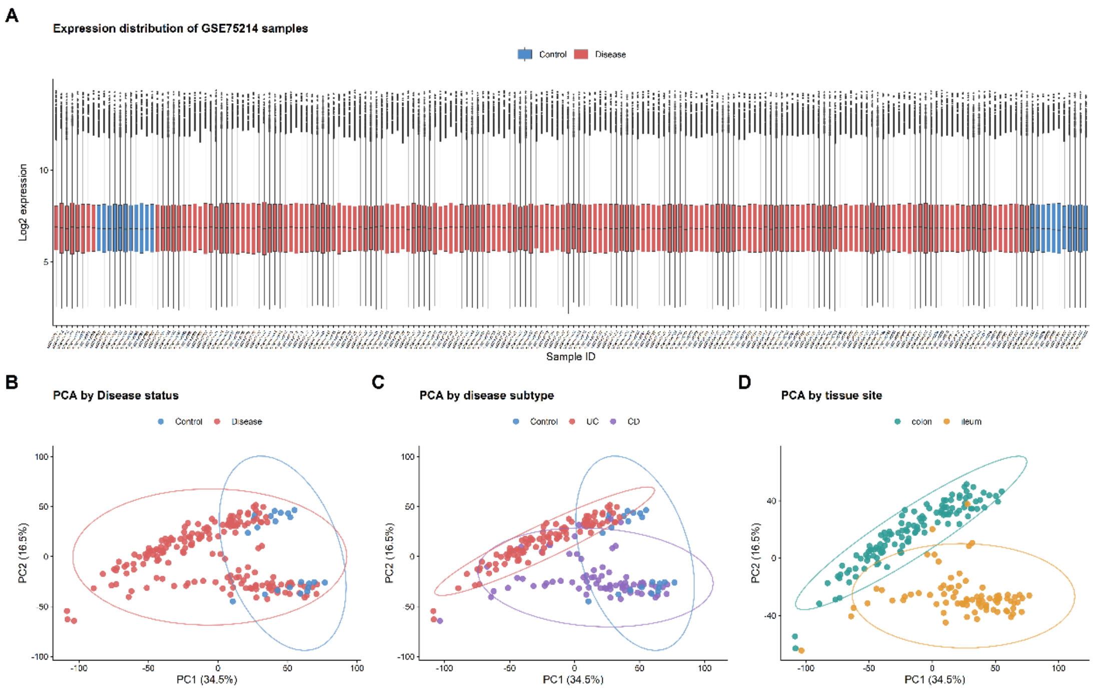

Figure S1. Quality assessment and principal component analysis of the GSE75214 cohort. (A) Expression distribution boxplot of all retained samples, colored by Control and Disease groups. (B) Principal component analysis (PCA) of Control and Disease samples. (C) PCA of samples stratified by disease subtype, including Control, ulcerative colitis (UC), and Crohn’s disease (CD). (D) PCA of samples stratified by tissue site, including colon and ileum.

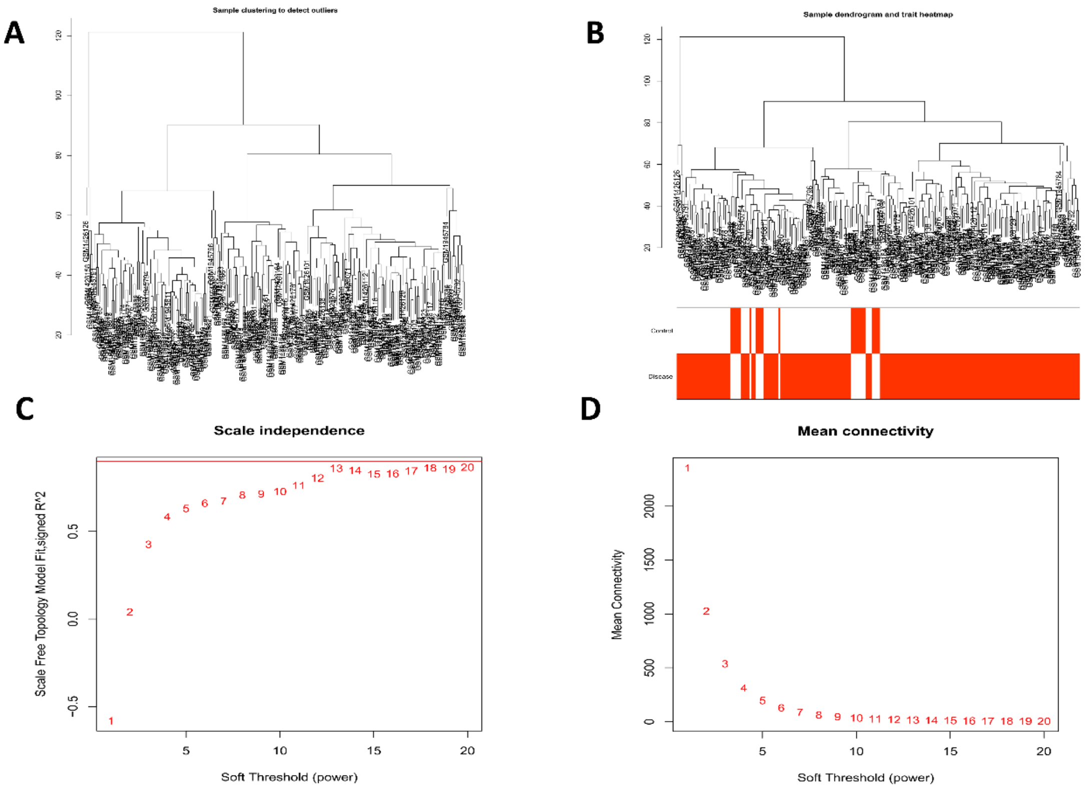

Figure S2. WGCNA sample assessment and soft-thresholding power determination. (A) Hierarchical clustering dendrogram of GSE75214 samples for outlier detection. (B) Sample dendrogram combined with Control/Disease trait annotation. (C) Scale-free topology model fit across different soft-thresholding powers. (D) Mean connectivity across different soft-thresholding powers. These analyses were used to determine the appropriate soft-thresholding power for WGCNA network construction.

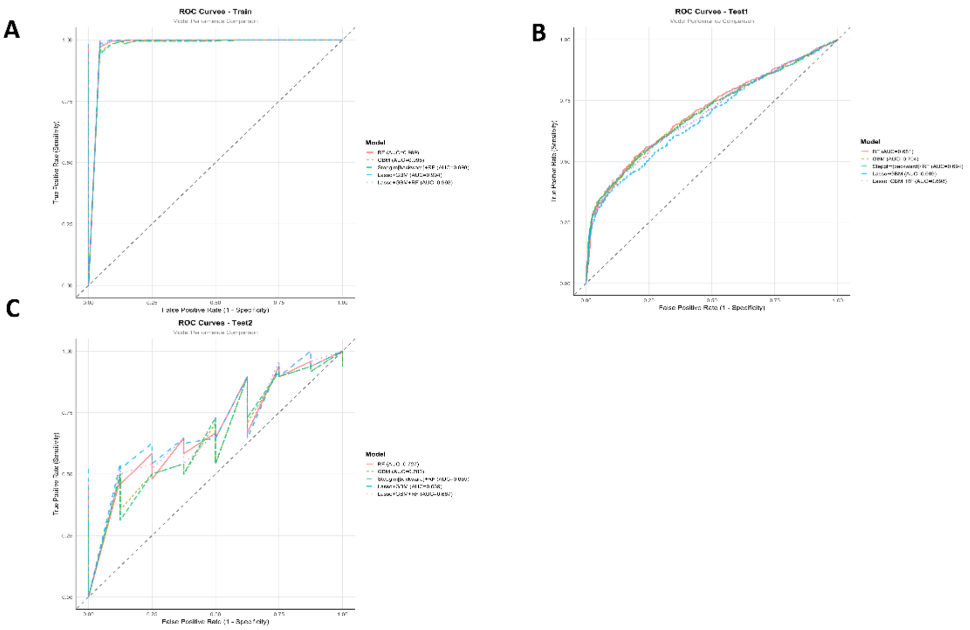

Figure S3. ROC curves of representative machine-learning models across the model-development and validation cohorts. (A) ROC curves of the top-ranked machine-learning models in the model-development cohort GSE75214. (B) ROC curves of the same models in the external validation cohort GSE193677. (C) ROC curves of the same models in the external validation cohort GSE235236.

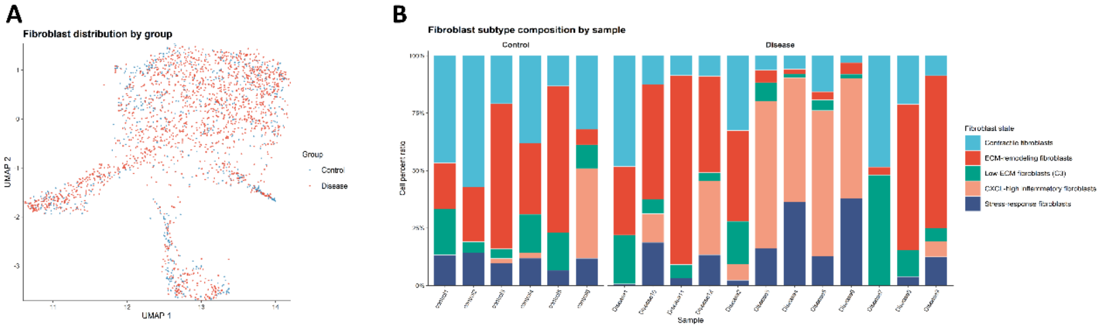

Figure S4. Fibroblast distribution and sample-level subtype composition. (A) UMAP distribution of fibroblasts colored by Control and Disease groups. (B) Sample-level composition of five fibroblast states in Control and Disease samples.
